# Prevalence of depression among medical students in Africa: Systematic review and meta-analysis

**DOI:** 10.1371/journal.pone.0312281

**Published:** 2024-12-26

**Authors:** Chilot Kassa Mekonnen, Hailemichael Kindie Abate, Zerko Wako Beko, Alebachew Ferede Zegeye, Abere Woretaw Azagew

**Affiliations:** Department of Medical Nursing, School of Nursing, University of Gondar, Gondar, Ethiopia; Madda Walabu University, ETHIOPIA

## Abstract

**Introduction:**

Depression has become a major health problem that students in a University encounter during their study life. At least one-third and possibly up to one-half of medical students show some form of psychological distress during their medical school. Aggregated evidence is scarce in Africa though there are published articles with various outputs. Therefore, this systematic review and meta-analysis aimed to pool those outputs to draw concert information crucial for devising strategies to tackle depression among students at the University.

**Objective:**

To determine the pooled prevalence of depression among African medical students.

**Method:**

Original articles about the prevalence of depression among African medical students were searched through known and international databases (PubMed, Scopus, Web of Science, and Cochran Library) and search engines (Google and Google Scholar). Data were extracted using a standard data extraction checklist that was developed according to Joanna Briggs Institute (JBI). The I^2^ statistics were used to identify heterogeneity across studies. Funnel plot asymmetry and Egger’s tests were used to check for publication bias. A Random effect model was used to estimate the pooled prevalence of depression among medical students in Africa. Statistical analysis was conducted using STATA version 11 software.

**Result:**

A total of 31 cross-sectional observational studies which provided information about the prevalence of depression among medical students were included in this systematic review and meta-analysis. The overall pooled prevalence of depression among medical students in Africa was 38.80% [95%CI (29.55, 48.05). Being a female medical student was [AOR = 0.25, 95%CI (0.15, 0.42)] and being a second-year medical student was [AOR = 0.26, 95%CI (0.10, 0.68)] times less likely to have depression.

**Conclusion:**

Depression affects well over one-third of medical students in Africa. Moreover, this systematic review and meta-analysis revealed that female medical students were less likely to develop depression. Therefore, this result suggested that medical schools or Universities and concerned authorities better offer possible early detection and prevention programs as per the magnitude. Furthermore, there has to be further research to figure out the potential factors perhaps using both qualitative and quantitative research approaches.

## Introduction

Depression is a common and serious mental disorder affecting well above 350 million people globally and is characterized by sadness, loss of interest or pleasure, feelings of guilt or low self-worth, disturbed sleep or appetite, feelings of tiredness and poor concentration [1,2].

It is differentiated from normal mood changes by the extent of its severity, symptoms, and duration of disturbance [3].

Depression has become a major health problem that students in a University encounter during their study life. At least one-third and possibly up to one-half of medical students show some form of psychological distress during medical school [4,5]. The time students join the University overlaps with an acute growing era and the time when many common mental disorders are about to happen. This resulted from existence changes such as departure from the household or family and usual linkages, a different setting, and comprehensive academic pressure, which is expected to complement augmented rates of mental problems such as depression [6,7].

This mental health problem is associated with poor academic performance, disability, and poor quality of life [8,9]. Years of stay in medical school is a unique period characterized by a transition period from adolescence to adulthood, and it is also the most crowded period which causes students to be anxious and depressed [10,11]. In this environment, students might face different unexpected problems that can have a negative consequence on their academic performance [12]. The studies in Bahrain [13], Portugal [14], and Nepal [15] showed that the prevalence of depression among medical students was 40.0%, 21.5%, and 29.2% respectively. The other studies revealed that the prevalence of depression among medical students was 33.0% in Iran with 28.0% Versus 23.0% for males and females [16], 41.2% in Brazil [17], and 70% in Pakistan [5]. Furthermore, it was 66.8% in China [8], 65.0% in Egypt [18], and 51.3% in Addis Ababa, Ethiopia [19]. Although there are pretty sizable articles done about depression among African University Medical students reports were not consistent and enough to generate a shred of rigorous evidence about this segment population so far. Furthermore, there is inconsistency among studies conducted amongst the countries and even within the countries which confused the reader to extract consistence evidence since different outcome measuring tools were utilized.

On top of the above variation in the prevalence of depression, this systematic review and meta-analysis were conducted to have pooled evidence for the scientific community at large. Besides, the result of this review study will have significant input on decisions and policymakers on a large scale regarding University medical students in Africa. Therefore, this study assessed the pooled prevalence of depression among medical students at African Universities.

## Research questions

What is the prevalence of depression among medical students in African public Universities?

### Inclusion and exclusion

#### Participants (population) of the Review

The review incorporated primary articles from Africa given the population are University medical students with a diagnosis of depression.

#### Condition of the review

In this review study, the prevalence of depression among medical students at the University was considered as the condition. Regarding this particular review, depression is defined as a depressed mood and loss of interest or pleasure in daily activities for long periods. But, not the regular mood changes and feelings about every day [20].

#### Context

African public Universities: For the sake of this particular review, the African public University was defined as any public university which delivered medical education in African countries [21].

*The outcome of interest*. This review aimed to determine the pooled prevalence of depression among medical students in Africa. The targeted articles were observational cross-sectional studies that correctly reported the outcome of interest (depression) among medical students.

## Method

### Protocol registration and reporting

The current systematic review has utilized the guidelines developed by the Joanna Briggs Institute (JBI) for Systematic Reviews [22] and the report is written consistent with the revised 2020 PRISMA guidelines [23]. This systematic review and meta-analysis title and its protocol were registered in the PROSPERO online database (with registration number CRD42023431163). The result of this review presentation was consistent with the standard preferred Reporting Items [23] for the Systematic Review and Meta-analysis (PRISMA) checklist (S1 File).

### Searching strategies

The known and international databases (PubMed, Scopus, Web of Science, and Cochran Library) and search engines (Google and Google Scholar) were used to locate research articles on the prevalence of depression among African medical students. The string for searching was developed using “AND” and “OR” Boolean operators with the keywords extracted from the Medical Subject Headings (MeSH) database. The search strategy was based on the research question of this review and utilized the **CoCoPop (Co =** Condition**, Co =** Context**, Pop =** Population**) model.** The article locating strategy was through depression OR "depression prevalence" OR "depression magnitude" OR "depression epidemiology" OR "depressive symptoms" OR "depressive disorder" OR "major depressive disorder" AND "medical students" OR "undergraduate medical students" OR "Bachelor’s degree medical students" OR "doctorate medical students" AND "University students" OR "Public university students" OR Africa. This search strategy primarily aimed to trace all reviewed (published) and unpublished primary studies. The list of all retrieved primary articles and systematic review and meta-analysis references were also screened or cross-referenced to get extra studies. The sources of information range from electronic databases to direct contact with the principal investigator if mandatory. The first search through Pub Med, Cochran Library, Scopus, Web of Science, Google, and Google Scholar was done in April 2023. The final search for updating was conducted from May 05/ 2023 to June 6/ 6/2023. The publication date was used as a filter mechanism in which articles published from January 2013 to May 2023 were included in the current systematic Review and Meta-analysis study to generate the most recent evidence for the scientific community.

### Inclusion and exclusion criteria

**Inclusion criteria**: Articles were included if and only if they fulfil the following predetermined criteria Published in English, Conducted in Africa, Observational studies (both analytical and descriptive cross-sectional), Articles with clear outcomes about depression, and published from 2013 to 2023 were included in this particular systematic review and Meta-analysis.

**Exclusion criteria**: after a thorough examination of the titles and abstract using eligibility criteria, those articles failed to give the aggregate prevalence of depression among medical students, provide insufficient information about the outcome of interest(depression), interventional studies, case reports, case series, and commentaries, and studies did not use standardized and validated depression measuring tools were excluded.

### The study selection and outcome

After comprehensive searching, all located citations were selected and exported to Endnote citation manager software version X7. Following this, irrelevant and duplicated articles were removed. Then two independent researchers (HKA and AWA) screened each particular article for its title, abstract, and full text by far and cross-checked it against the inclusion criteria. The other research team (AFZ and ZWB) checked the screened articles with full text for details under already defined criteria to take it to the final review process. Any sort of disagreement between the research team while including and excluding articles on predefined criteria of this particular review was resolved by a thorough discussion of the team. The exclusion of the articles was presented with countable reasons which could be consistent with the pre-defined criteria. The result of searching further screening and inclusion process of articles in this review was done in agreement with the PRISMA guidelines for Systematic Review and Meta-analysis 2020.

### Quality appraisal of included studies

The methodological quality of the included studies was critically appraised by three independent reviewers (AWA, CKM and HKA) by using the standardized JBI critical appraisal tool for the studies reporting prevalence [22]. This critical appraisal tool consists of 9 items designed to oversee the study population, sample size adequacy of the study, study subject and setting, reliability of condition or problem measurement, the appropriateness of the Statistical test used to analyze the data, and adequacy of the response rate of each selected article for the review process (S3 File). Each item has an answer of “No”, “Yes” or “unclear” to rate its risk of bias (ROB). After the critical appraisal, the reviewers decided to include or exclude screened articles based on the overall quality of the appraisal score in which those articles with the lowest score out of 9 considered as poor quality or high risk of bias. The article was prone to exclude when the score was below average that is 4.5 of the three independent reviewers. In this regard, there had to be more than 3 “No” or unclear” quality categories for the article to be excluded from the review. This critical appraisal threshold was supported by a previously published systematic review and meta-analysis study [24]. Any sort of disagreement between the involved reviewers was solved through the discussion of the reviewers. Furthermore, if the disagreement unfolds, the fourth reviewer was indicated to oversee the source of the doubt and reach to consensus.

### Data extraction

Data were independently extracted by four authors using a standardized data extraction format that was developed according to the 2014 Joanna Briggs Institute Reviewers’ Manual [25]. The tool includes Authors, country, and y year, study design, sample size, the prevalence of depression among medical students, tool used to measure the outcome, response rate, and risk of bias assessment score included in the extraction (**S2 File**). The data were extracted by two independent reviewers and any inconsistent data was cross-checked. The disagreement between the reviewers was solved by a thorough discussion.

### Outcome measurements

The primary outcomes of this meta-analysis and systematic review were the pooled prevalence of depression among medical students elsewhere in Africa.

### Data synthesis and analysis

The outcome of included primary studies was narratively presented and expanded with supplementary materials in text, tables, and figures where necessary. All necessary and relevant information from every article was extracted through a Microsoft Excel spreadsheet, and exported to STATA Version 11 for further analysis. The random-effects model was employed to estimate the pooled effect size of depression among medical students due to the presence of heterogeneity. Heterogeneity was identified by using the standard chi-square and I-square statistical tests. The variation between different study characteristics such as the country where the primary article was conducted and the outcome ascertainment tool was investigated through subgroup analyses. These subgroup analyses could demonstrate the sources of heterogeneity and let the researcher for another remedy such as the use of meta-regression to treat this heterogeneity. Moreover, the publication bias was assessed through visual inspection of the funnel plot, Begg- Mazumdar Rank correlation tests and Egger’s test to see the funnel plot’s asymmetry. The influence of individual articles on the overall pooled effect size estimate or the prevalence of depression was assessed by using sensitivity analysis. The forest plot with 95% CI was used to present the overall pooled prevalence as well as the subgroup pooled prevalence of depression among medical students in Africa.

## Result

### The article selection and outcome

**Fig 1:**
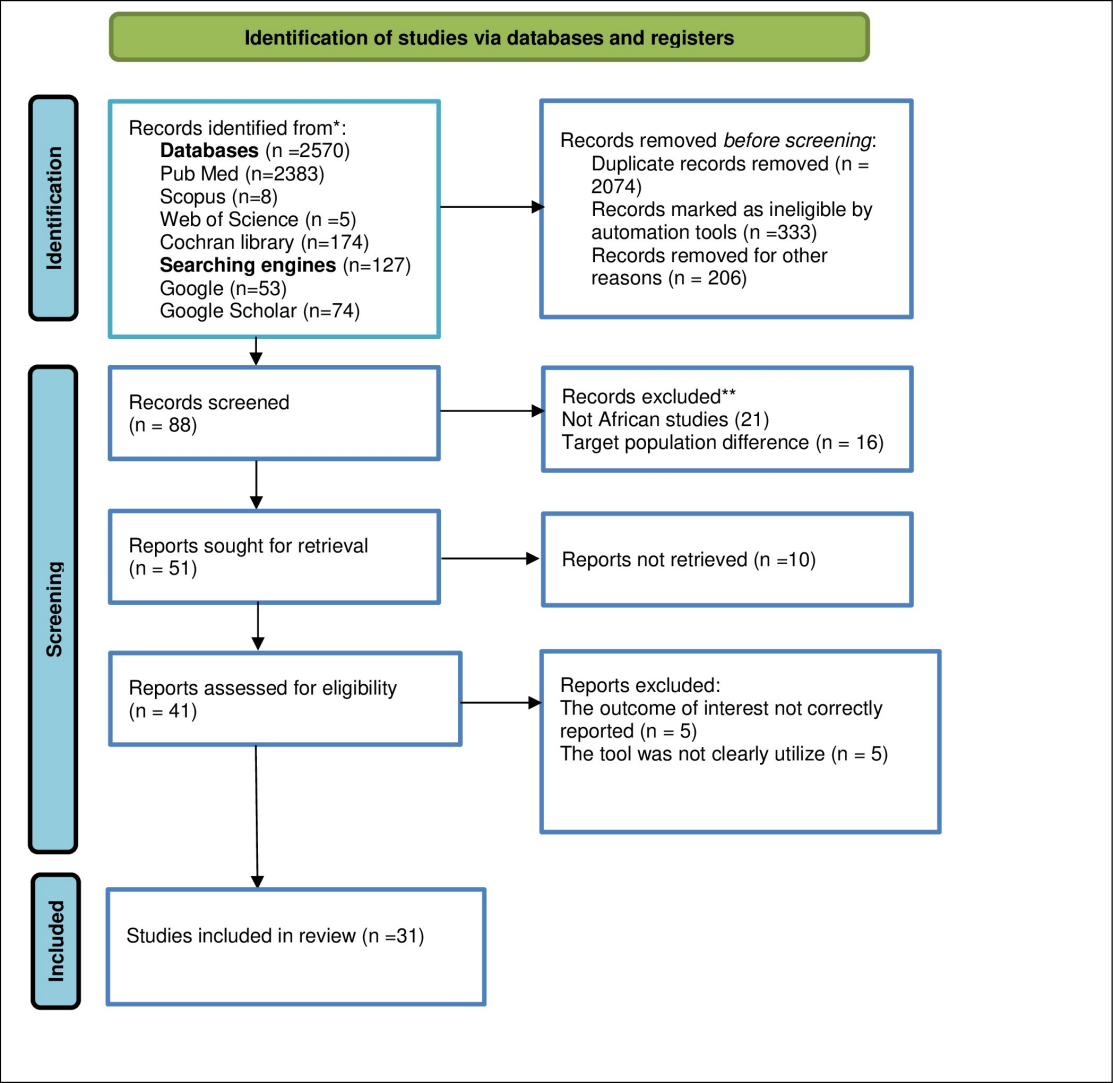
PRISMA flow diagram of the included studies.

In this systematic review and meta-analysis study a total of 2697 articles related to the prevalence of depression in African Medical students were identified using electronic databases and search engine websites. Among overall articles found 2074 were removed for being irrelevance and duplicated and the other pretty sizable articles were removed for not being ineligible (study design and Title difference) by automation tools and other reasons (333 vs. 20) respectively. The remaining 88 articles were eligible for screening. Of these screened 37 papers were excluded due to the region of study or not being conducted in Africa and target population difference (those articles conducted among undergraduate University students). With further screening, 51 articles were sought for retrieval and 9 were not retrieved for one and the other reason. Moreover, 42 research articles were assessed for eligibility to be included in the review process, but with the outcome of interest and measurement tool ambiguity a total of 10 articles were excluded. Finally, 31 original research articles were incorporated in the final systematic review and meta-analysis (**Fig 1**).

### The methodological quality assessment of included studies

There are a total of 31 articles assessed for methodological quality using a 9-point score tool developed by JBI for observational prevalence studies. The outcome of the quality appraisal ranged from moderate to high methodological quality in which eleven studies [26–36] scored 9 points, eleven studies [19,37–46] scored 8 points, four studies [18,47–49] scored 7 points and the remaining five studies [50–54] scored 6 points (S3 **File**).

### Characteristics of the included primary studies

In this systematic review and meta-analysis, 34,189 participants were included with a response rate of 100%. The studies included in this review were observational cross-sectional studies published from 2016 to 2023. The smallest sample size was 92 from the study conducted in Morocco [43] followed by the studies 170 in Libya [52] and 203 in Morocco [38]. On the other hand, the largest sample size was 1058 from Sudan [46] followed by 1300 from Libya [39]. The prevalence of depression among medical students ranges from 10.10% to 75% from the studies in Nigeria and Sudan respectively [32,46]. The articles utilized five different types of outcome ascertainment tools such as HADS, BID, SRQ-20, PHQ-9, and DASS-21. In this regard 8(25.6%) studies used PHQ measuring tools, and 7(22.6%) studies used BID and DASS-21 measuring tools to ascertain depression among medical students. Regarding the risk of bias assessment, we have used JBI guidelines for observational prevalence studies with 9 9-point score. The quality score range was 6–9 among the included studies for this systematic review and meta-analysis (Table 1).

**Table 1:** Summary of the prevalence of depression among thirty-one studies of African medical students included in the systematic review and meta-analysis.

Author(Publication year)	Country	Tool used	study design	Population	sample	Prevalence	Response rate	Quality
Kebede et al., 2019 [19]	Ethiopia	HANDS	Cross-sectional	Medical students	273	51.30	98.5%	8
Dagnew et al., 2020 [37]	Ethiopia	BDI	Cross-sectional	Medical students	383	34.73	97.7%	8
S van der Walt et al., 2020 [26]	South Africa	HANDS	Cross-sectional	Medical students	473	25.00	100%	9
Bawo O. James et al.,2017 [27]	Nigeria	HANDS	Cross-sectional	Medical students	623	21.30	98.1%	9
Joshua Falade et al., 2020 [28]	Nigeria	HANDS	Cross-sectional	Medical students	944	14.30	97.8%	9
M. Barrimi et al. 2020 [51]	Morocco	BDI	Cross-sectional	Medical students	605	10.40	100%	6
Mboya et al., 2020 [38]	Tanzania	SRQ-20	Cross-sectional	Medical students	203	14.3	100%	8
Olum et al., 2020 [47]	Uganda	PHQ-9	Cross-sectional	Medical students	331	21.50	93.8%	7
Ngasa et al., 2017 [29]	Cameroon	PHQ-9	Cross-sectional	Medical students	618	30.60	90.4%	9
Njim T, et al., 2019 [40]	Cameroon	PHQ-9	Cross-sectional	Medical students	413	66.34	82.6%	8
Edmund Ndudi Ossai et al., 2021 [35]	Nigeria	BDI	Cross-sectional	Medical students	522	26.6	100%	9
El-Gilany et al., 2019 [31]	Egypt	BDI	Cross-sectional	Medical students	900	25.20	100%	9
C. E. NWACHUKWU ET AL., 2021 [32]	Nigeria	HANDS	Cross-sectional	Medical students	690	10.10	100%	9
Mohamed Fawzy et al., 20117 [18].	Egypt	DASS-21	Cross-sectional	Medical students	700	65.00	100%	7
Narushni Pillay et al., 2016 [50]	South Africa	DASS-21	Cross-sectional	Medical students	230	15.6	Not stated	6
Uzoechi Eze Chikezie et al., 2021 [48]	Nigeria	DASS-21	Cross-sectional	Medical students	243	25.5	100%	7
Wafaa et al., 2020 [33]	Egypt	DASS-21	Cross-sectional	Medical students	390	45.1	100%	9
Sherif RF et al., 2021 [52]	Libya	PHQ-9	Cross-sectional	Medical students	170	45	100%	6
Suraj, et al., 2021 [41]	Nigeria	SRQ-20	Cross-sectional	Medical students	279	15.1	100%	8
Leta Melaku et al., 2021 [34]	Ethiopia	DASS-21	Cross-sectional	Medical students	260	53	98.1%	9
Tarteel Musa et al., 2022 [53]	Sudan	HANDS	Cross-sectional	Medical students	355	78	100%	6
Khalid A. Khalil et al. [39]	Libya	PHQ-9	Cross-sectional	Medical students	1300	45	74.6%	8
H Essangri et al., 2021[42]	Morocco	BDI	Cross-sectional	Medical students	549	74.7	100%	8
Rammouz et al., 2023 [43]	Morocco	BDI	Cross-sectional	Medical students	92	41.3	91.4%	8
Shereen Esmat et al., 2021 [44]	Egypt	BDI	Cross-sectional	Medical students	238	38.2	79.3%	8
Mwita M et al., 2020 [45]	Tanzania	PHQ-9	Cross-sectional	Medical students	353	41.36	100%	8
Sserunkuuma, J., et al., 2023 [36]	Uganda	PHQ-9	Cross-sectional	Medical students	269	16.73	100%	9
S. H. Mustafa et al., 2022 [56]	Sudan	SRQ-20	Cross-sectional	Medical students	432	55.8	100%	9
Mohamed, E.A.A., et al., 2018 [49]	Sudan	PHQ-9	Cross-sectional	Medical students	440	67	100%	7
Dafaalla, M., et al., 2016 [54]	Sudan	DASS-21	Cross-sectional	Medical students	487	53.4	97.4%	6
Nubi et al., 2022 [46]	Sudan	DASS-21	Cross-sectional	Medical students	1058	75	99.9%	8

## Prevalence of depression among medical students

**Fig 2:**
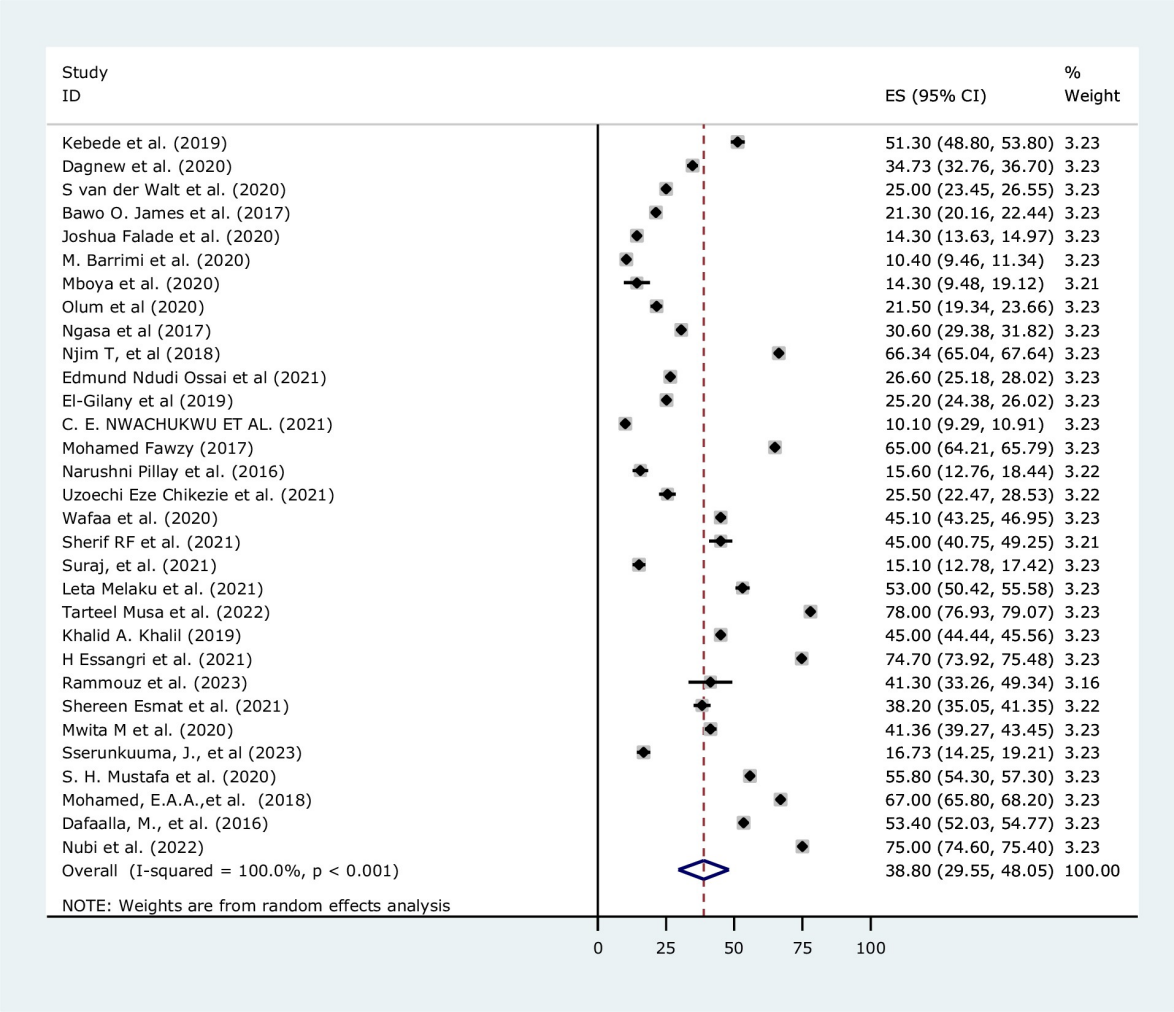
Forest plot of the prevalence of depression among medical students in Africa.

A total of 31 primary articles were appraised and retrieved to pool the prevalence of depression among medical students in Africa. These articles were from ten African countries with Nigeria six[27,28,30,32,41,55], Sudan five[46,49,54,56,57], Egypt four[18,31,33,44], Morocco three [42,51,58], Ethiopia three [34,59,60], and two from South Africa [26,50], Tanzania [38,61], Uganda [36,47], Cameroon [29,40], and Libya [52,62] each (Table 1). The prevalence ranged from 10.10% to 75.0% in Nigeria and Sudan respectively [32,46]. The pooled prevalence of depression among medical students was 38.80% [95%CI (29.55, 48.05), I^2^ = 100.00%, P<0.001]. The effect size of the overall pooled prevalence of depression among medical students was presented using a forest plot (**Fig 2).**

### Determinants of depression among medical students

In this systematic review and meta-analysis study we have found variables such as sex of students, year of study, academic stage(preclinical and clinical), and social support reported as predictors of depression in our review of primary articles. Then we have pooled twelve [26,29,31–34,41,44,47,51,59,61] studies reporting being female as a predictor of depression and the overall pooled effect size with a fixed-effects model for being a female medical student was [AOR = 0.25, 95%CI (0.15, 0.42), I^2^ = 0.0%, p-value = 0.65]. Besides, we have pooled five [34,41,47,59,61] studies that reported year of study as a factor for depression and found the overall pooled effect size for being a second-year medical student was [AOR = 0.26, 95%CI (0.10, 0.68), I^2^ = 0.0%, P-value = 0.96] **Table 2**.

**Table 2:** Determinant factors of depression among medical students in Africa.

Determinants	Categories	Pooled Effect size	I-squared (%)	Sample	P-value
Sex Twelve studies [26,29,31–34,41,44,47,51,59,61]	Male	1	1		1
	female	0.25(0.15, 0.42)*	0.0	4720	0.65
Year of Study					
Five studies [34,41,47,59,61]	First-year	1.86(0.03, 11.20)	0.0	1496	1.00
	2nd year	0.26(0.10, 0.68)*	0.0	1496	0.96
	3rd year	0.40(0.11, 1.44)	0.0	1496	0.99
	4th year	0.50(0.13, 1.95)	0.0	1496	0.99
	5th year	0.76(0.44, 1.29)	1.15	1496	0.89
Academic stage					
Four studies [26,29,31,32]	Preclinical	1	1		1
	Clinical	0.78(0.19, 3.16)	0.0	2681	0.97
Social support					
Three studies [38,41,59]	Yes	1			
	No	1.46(0.06, 5.27)	0.0	954	0.99

### Heterogeneity assessment

In this systematic review and meta-analysis, the analysis output using a random-effects model showed high variability across the primary articles included in the study (I^2^ = 100%, P<0.001). This variability is inevitable in meta-analysis studies resulting from quality differences of the included studies, methodological differences, sample size, inclusion and exclusion, and the difference in measuring tools to ascertain the outcome of interest. Therefore, we have conducted the meta-regression analysis by using publication year, sample size, and standard error as covariates to figure out the potential source of heterogeneity among included studies. In this regard, the meta-regression analysis revealed that no significant correlation was found between the outcome of interest (depression) and the included covariates by far (p = 0.662 for publication year and P = 0.686 for sample size). Hence, there was no statistically significant association and possible existence of variability as shown (Table 3). This again implies that the source of high variability (heterogeneity) could be due to chance or the other variables not investigated in this particular review.

**Table 3:** Meta-regression analysis to see between studies variation (heterogeneity).

Sources of heterogeneity	Coefficients	Std. Err	P-value
**Publication year**	**.0252903**	**.0572149**	**0.662**
**The sample size of the original article**	**.0001321**	**.0003229**	**0.686**

### Subgroup analysis

**Fig 3:**
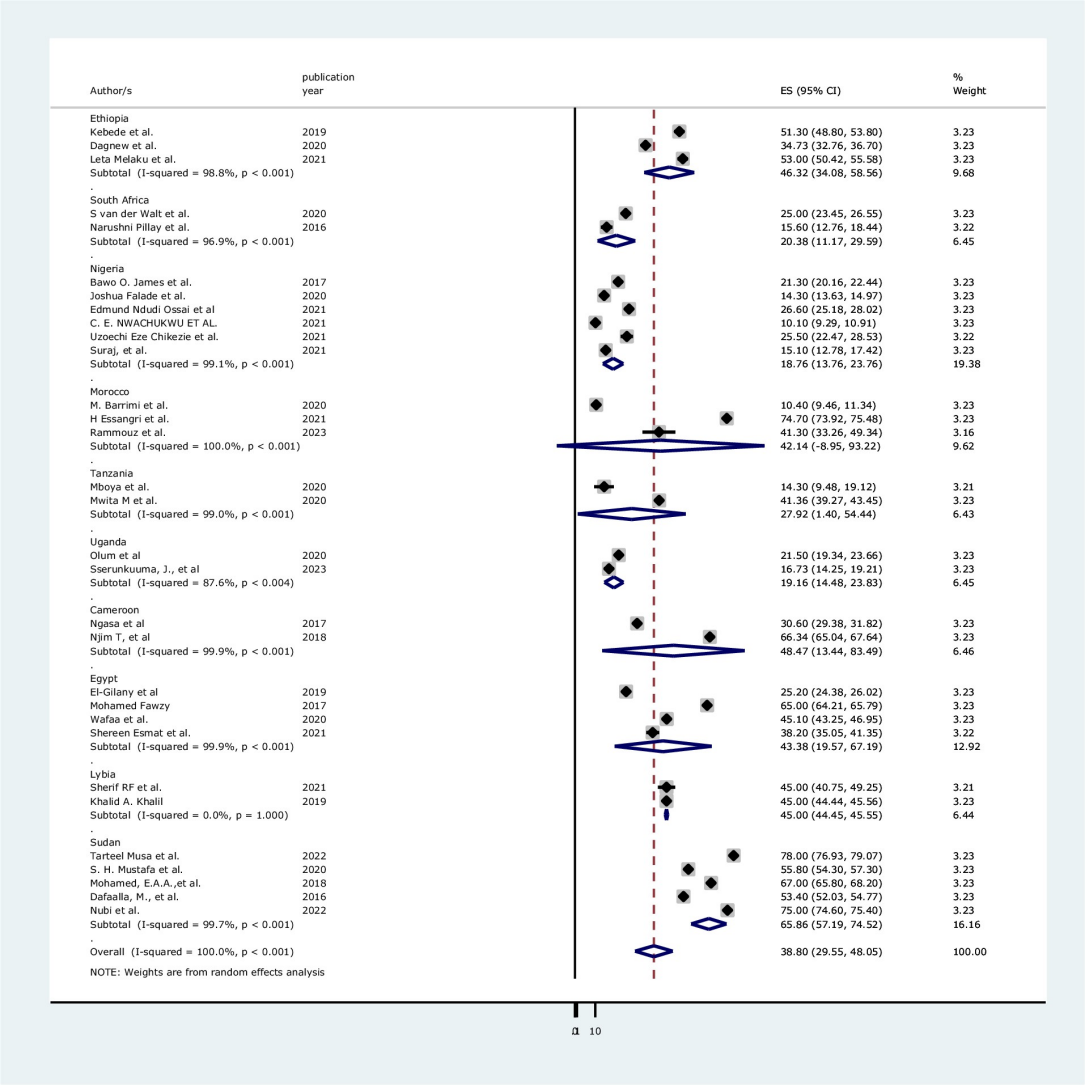
The Forest plot of subgroup analysis by country of 31 included studies.

In this review, the subgroup analysis was employed by using the country and the outcome ascertainment tool used in the primary reviewed articles. In the subgroup analysis by country the lowest pooled prevalence using the random-effects model was 18.76% [95%CI (13.76, 23.76), I^2^ = 99.1%, p<0.001] in Nigeria followed by 19.16% [95%CI (14.48, 23.83), I^2^ = 87.6%, p<0.004] in Uganda. Whereas, the highest pooled prevalence was found at 65.86% [95% CI (57.19, 74.52), I^2^ = 97.7%, p<0.001] in Sudan followed by 48.47% [95%CI (13.44, 83.49), I^2^ = 99.9%, p<0.001] in Cameroon (Fig 3).

**Fig 4:**
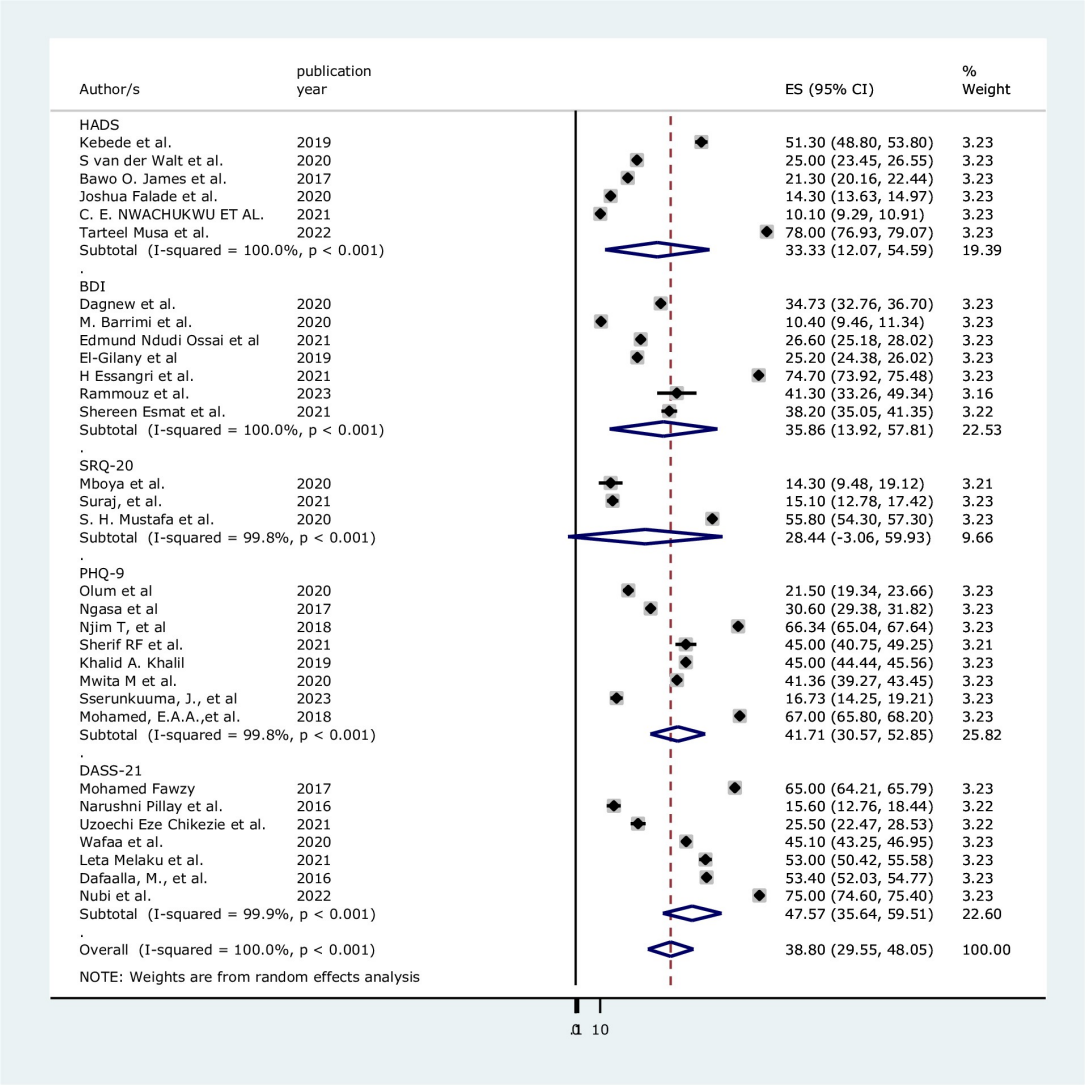
Forest plot analysis of the subgroup by the outcome measurement tool.

Moreover, in the subgroup analysis using the outcome measuring tool the highest pooled prevalence found to be 47.57% [95%CI(35.64, 59.51), I^2^ = 99.9%, p<0.001] with outcome measuring tool of SRQ-20 followed by 41.71%[95%CI(30.57, 52.85), I^2^ = 99.8%, p<0.001] using PHQ-9 measuring tool for the diagnosis of depression among medical students in Africa (Fig 4).

### Sensitivity analysis

**Fig 5:**
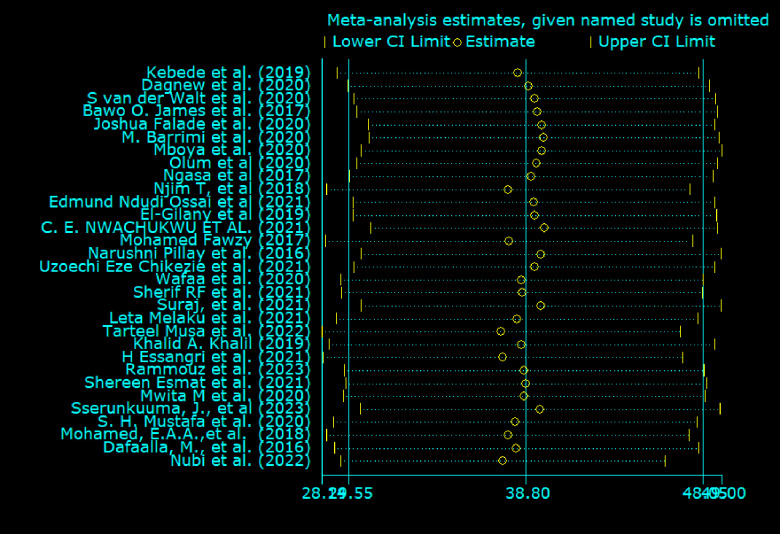
Sensitivity analysis of the included studies.

We have conducted a sensitivity analysis to identify whether there is evidence of the influencing effect of one study on the other. The output of leave-one-out sensitivity analysis through the random-effects model revealed that there was no individual study that influenced the overall pooled estimate of depression among medical students in this particular review. For every single study, the effect size indicated relates to the overall pooled effect size generated from meta-analysis omitted that particular study **(Fig 5**).

### Publication bias assessment

**Fig 6:**
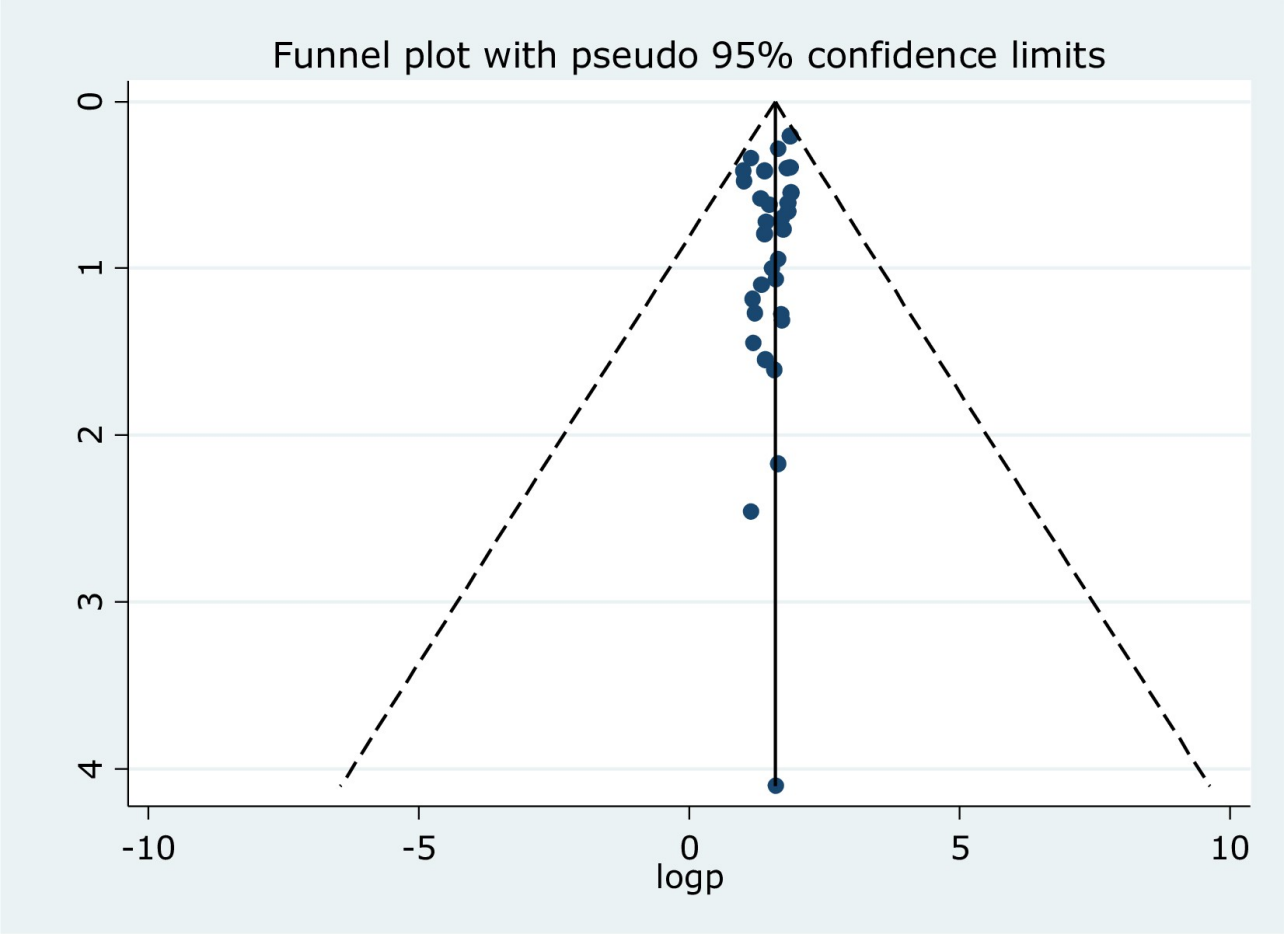
Funnel plot of the included studies to show asymmetry.

In this particular systematic review and meta-analysis study publication bias was assessed by using visual inspection of a funnel plot which referred to asymmetry, as sixteen studies lay to the left and eleven studies were to the right of the line (Fig 6). However, it was not statistically significant as shown in Egger’s test (P = 0.221) which depicted that the outputs were not influenced by publication bias for one or the other reasons. It is evidenced that the asymmetry of the funnel plot is not always related to publication bias [63].

## The included studies certainty assessment evidence

In this systematic review and meta-analysis study, all the included primary articles were cross-sectional and we reached at low certainty of evidence conclusion and we extend our assessment of certainty of the evidence through five down grading domains and three up grading elements of the GRADE certainty assessment criteria [1]. In this regard, evidences liable to inconsistencies because of a few studies were eligible in this particular study. This was revealed by the existence of heterogeneity in the estimate of the outcome of interest across different regions of the continent Africa. However, we had found no publication bias and risk of bias as most included studies followed robust and scientific methods. The absence of publication bias revealed by the egger’s test and funnel plots. The absence of outcome measurement imprecision was revealed by well enough sample size of all included studies and narrow confidence interval of the pooled effect size. Besides, no included studies indirectly measured the outcome of interest (prevalence depression) among medical students, but direct measurement was employed from all the included studies. The confounding were controlled through seeing all the potential factors that probably disturb the measurement of estimate of the outcome variable and the estimate of outcome of interest in all included studies was well enough. Overall, the prevalence of depression among medical students in Africa is highly liable to change given more primary articles included; therefore it entails further studies to produce robust evidence that authors could rely on for decision making capability and purpose **S1–S3 Tables.**

## Discussion

This systematic review and meta-analysis were conducted to determine the pooled prevalence of depression and its association among medical students in Africa. The overall pooled prevalence of depression among African medical students was 38.80% [95%CI (29.55, 48.05), I^2^ = 100.00%, P<0.001]. The current meta-analysis result is consistent with the studies conducted in China [64] and India [65] with a pooled prevalence of 37.9% and 40% of medical students experienced depression respectively. However, it was higher than the studies conducted in China and Spain [66–68] with a pooled prevalence of 27%, 29%, and 31% respectively. The possible explanation for the variation between the current study and Spain was the time gap and sample size difference in which the review in Spain was conducted only from articles published from 2019 to 2020 during the COVID-19 pandemic. Moreover, the current is higher than the study conducted in China with a pooled prevalence of 33.7% of the medical students experienced depression [69]. The possible discrepancy might be because there are socio-economic, sociocultural, number of reviewed articles, tool and time variations with the current systematic review and meta-analysis to that of China. There is also a time gap which accounts for the variation in the proportion of depression across the Nations.

It was also higher than the studies conducted in Singapore [70] 28.0%, and Brazil [71,72] 30.6% and 28.51% pooled prevalence of depression among medical students. Moreover, the findings of this systematic review and meta-analysis are higher than the studies conducted in America with a 27.2% pooled prevalence of depression [73], and in China [74] with a pooled prevalence of 27% among medical students. The possible variation might be because there is a population, socio-cultural, curricular and extracurricular differences in Asia and Africa. For instance, infrastructural differences for relaxation in African Universities might be quite different from European Universities [75]. Besides, there might be a variation in an organizational support system that contributes a lot to the well-being of students at that particular University. Furthermore; the current study finding is higher than the study conducted in China [76] with a pooled prevalence of 19.9%. The difference might be since the study in China was conducted only in one national University Medical Students and the outcome measuring tool was BID whereas the current study is over African Universities which might cause the discrepancy [77].

However, the current finding is lower than the study conducted in North America 66.5% of medical students experienced depression [66]. A possible explanation might be the review in North America included all articles published between the years 1906 to 2013 which caused sample variation, tool variation, and time gap. The finding was lower than the reviews conducted in China [67] and India [78] with the pooled prevalence of depression ranging from 13.10 to 76.21% and 50.% respectively. The possible explanation for the variation might be due to sociocultural variation, sample size difference and the review in India included only studies published between 2019 to 2020 that were during the COVID-19 pandemic which probably increased depression among medical students due to lockdown, long time social isolation, and perceived probability of encountering the pandemic. This has been supported by the study conducted in Bahrain [79]. Furthermore, we have pooled twelve [26,29,31–34,41,44,47,51,59,61] studies reporting being female as a predictor of depression and the overall pooled effect size with a fixed-effects model for being a female medical student was 75% [AOR = 0.25, 95%CI (0.15, 0.42)] less likely to have depression as compared to being male. This was consistent with the study conducted in [16]. Similarly, we have pooled five [34,41,47,59,61] studies that reported year of study as a factor for depression and found being a second-year medical student was 76% [AOR = 0.26, 95%CI (0.10, 0.68)] less likely to experience depression as compared to its counterparts. This might be because students are adapting to the new learning environment and other academic-related variables such as teaching and learning processes, the evaluation techniques, even though this is the time that students are becoming familiar with medical term knowledge. Therefore, as the level of study increases the fear, stress, anxiety and depression level decreases [11,80]. Besides, the current systematic review and meta-analysis were conducted in Africa which is a continent with articles from 10 countries, but the above reviews were conducted in individual countries which could be a reason for the discrepancy. The output of this systematic review and meta-analysis provides an up-to-date body of knowledge about the magnitude and severity of depression among medical students in Africa which is an alarming aggregated and rigorous evidence to think about and devise strategies to tackle it. Moreover, the finding has had an important clinical implication in providing pooled evidence of depression for those who are interested in the area, particularly for those focusing on mental health and well-being. Furthermore, this study has its strength in generating pooled data in Africa which could help those in need of such evidence to devise strategies to tackle depression. However, it has also important limitations such as it only includes articles published in English, and the primary studies were all observational cross-sectional studies. In addition, this meta-analysis didn’t include the factors attributed to depression among medical students and only included the studies conducted in Africa. Therefore; further global-based studies to assess other factors related to depression ought to be done

## Conclusion

This systematic review and meta-analysis revealed that well over one-third of medical students in Africa are affected by depression during their duration. Furthermore, being a female medical student and being a second-year medical student were negatively associated with depression. But, in other words, being a male medical student was highly likely to develop depression. Moreover, there has to be further research to figure out the potential factors perhaps using both qualitative and quantitative research approaches. Therefore, this result suggested that medical schools or Universities and concerned authorities better offer possible early detection and prevention programs as per the magnitude.

## Supporting information

S1 File PRISMA checklist. (DOCX)

S2 File Data extraction. (DOCX)

S3 File Quality appraisal checklist. (DOCX)

S1 Table All studies identified in the literature search, including those that were excluded from the final analyses. (DOCX)

S2 Table A table of all data extracted from the primary research sources for the systematic review and/or meta-analysis. (DOCX)

S3 Table Grade score of the included studies in the final systematic review and meta-analysis. (DOCX)

S1 Data (DOCX)

## Abbreviations

BDI: Beck Depression Inventory
CI: Confidence interval
COVID-19: Coronavirus 2019
DASS: Depression Anxiety Stress Scale
HADS: Hospital Anxiety and Depression Scale
PHQ: Patient Health Questionnaire
SNN: South Nation and Nationality Region
SRQ: Self Reporting Questionnaire
