# Supplementary material for: Prevalence of depression among medical students in Africa: Systematic review and meta-analysis
Source: PLoS One. 2024 Dec 26;19(12):e0312281. doi: 10.1371/journal.pone.0312281 (PMC11670985; doi:10.1371/journal.pone.0312281)
Supplement: S1 File — (DOCX) [file pone.0312281.s001.docx]

| **Section and Topic** | **Item #** | | **Checklist item** | **Location where item is reported** |
| --- | --- | --- | --- | --- |
| **TITLE** | | | |  |
| Title | 1 | | **Prevalence of depression in African Medical students: Systematic Review and Meta-analysis** |  |
| **ABSTRACT** | | | |  |
| Abstract | 2 | | **Abstract**  **Introduction:** Depression has become a major health problem that students in University encounter during their study life. At least one-third and possibly up to one-half of medical students show some form of psychological distress during medical school. In this environment, students might face different unexpected problems that can have a negative consequence on their academic performance. Aggregated evidence is scare in Africa though there are published articles with various outputs. Therefore, this systematic review and meta-analysis aimed to pool those outputs to draw concert information is crucial for devising strategies to tackle depression among students in at the University.  **Objectives:** To determine the pooled prevalence of depression among African medical students. **Method**: Original articles about the prevalence of depression among medical students in Africa were searched through known and international databases (PubMed, Scopus, Web of Science, and Cochran library) and searching engines (goggle and Google scholar). Data were extracted using a standard data extraction checklist that was developed according to Joanna Briggs Institute (JBI). The I^2^ statistics were used to identify heterogeneity across studies. Funnel plot asymmetry and Egger's tests were used to check for publication bias. A Random effect model was used to estimate the pooled prevalence of depression among medical students in Africa. Statistical analysis was conducted using STATA version 11 software.  **Result:** A total of 31 cross-sectional observational studies which provided information about prevalence of depression among medical students were included in this systematic review and meta-analysis. The overall pooled prevalence of depression among medical students in Africa was 38.80% [95%CI (29.55, 48.05).  **Conclusion:** The prevalence of depression among medical students in Africa was found to be high. Therefore, the respected Minister of Education, Health and respective Universities with other stakeholders better devise mechanisms to tackle depression through creating smooth teaching and learning environment.  **Keywords:** Prevalence; Depression; Depressive symptoms; mental health; Medical Students; African; University |  |
| **INTRODUCTION** | | | |  |
| Rationale | 3 | | Depression is the most common mental health problem that occurs all over the world, and this disorder affects an individual’s life [1]. Depression is a significant public health problem which is characterized by sadness, loss of interest in activities, and decreased mood. It is differentiated from normal mood changes by the extent of its severity, symptoms, and duration of disturbance [2]. Depression has become a major health problem that students in University encounter during their study life. At least one-third and possibly up to one-half of medical students show some form of psychological distress during medical school [3, 4]. These mental health problems are associated with poor academic performance, disability, and poor quality of life [5]. Years of stay in medical school is a unique period characterized by a transition period from adolescence to adulthood, and it is also the most crowded period which imposes students to be anxious and depressed [6, 7]. In this environment, students might face different unexpected problems that can have a negative consequence on their academic performance [8].  Although there are a pretty sizable articles done about depression in African University Medical students, the out puts are not consistent and enough to generate a rigorous evidence about this segment population so far.  On top of the above variation in the prevalence of depression, this systematic review and meta-analysis was conducted to have pooled evidence for the scientific community at large. Besides, the result of this review study will have a significant input for decision and policy makers in the large scale regarding University medical students in Africa. Therefore, this study assessed the pooled prevalence of depression among medical students at the African Universities |  |
| Objectives | 4 | | To determine the pooled prevalence of depression among African medical students. |  |
| **METHODS** | | | |  |
| Eligibility criteria | 5 | | **Inclusion criteria**: Articles were included if and only if they fulfill the following predetermined criteria such as Published in English, Conducted in Africa, Observational studies (both analytical and descriptive cross-sectional), Articles with clear outcomes about depression, and published from 2013 to 2023 were included in this particular systematic review and Meta-analysis. |  |
| Information sources | 6 | | The known and international databases (PubMed, Scopus, Web of Science, and Cochran library) and searching engines (goggle and Google scholar) were used to locate research articles on the prevalence of depression among medical students in Africa. |  |
| Search strategy | 7 | | The string for searching was developed using “AND” and “OR” Boolean operators with the keywords extracted from the Medical Subject Headings (MeSH) database. The search strategy based the research question of this review and utilized the **CoCoPop (Co=**Condition**, Co=**Context**, Pop=**Population**) model.**  The article locating strategy was through depression OR "depression prevalence" OR "depression magnitude" OR "depression epidemiology" OR "depressive symptoms" OR "depressive disorder" OR "major depressive disorder" AND "medical students" OR "undergraduate medical students" OR "Bachelor's degree medical students" OR "doctorate degree medical students" AND "University students" OR "Public university students" OR Africa. This searching strategy primarily aimed to trace all reviewed (published) and unpublished primary studies**.** The list of all retrieved primary articles and systematic review and meta-analyses reference were also screened or cross referenced to get extra studies. The sources of information range from electronic data bases to direct contact with principal investigator if mandatory. The first search through Pub Med, Cochran library, Scopus, Web of Science, Google, and Google scholar was done in April, 2023. The final search for updating was conducted from May 05/ 2023 to June 6/ 6/2023.Publication date was used as filter mechanism in which articles published from January 2013 to May 2023 included to the current systematic Review and Meta-analysis study to generate the most recent evidence to the scientific community. |  |
| Selection process | 8 | | After comprehensive searching, all located citations were selected and exported to Endnote citation manager software version X7. Following this, irrelevant and duplicated articles were removed. Then two independent researchers (HKA and AWA) screened each particular article for its title, abstract, and full text by far and cross check against the inclusion criteria. The other researcher team (AFZ and ZWB) checked the screened articles with full text for details in accordance to already defined criteria to take it to the final review process. Any sort of disagreement between research team while including and excluding articles on predefined criteria of this particular review was resolved by a thorough discussion of the team. Exclusion of the articles was presented with countable reasons which could be consistent with the pre-defined criteria. The result of searching further screening and inclusion process of articles in this review was done in agreement with the PRISMA guidelines for Systematic Review and Meta-analysis 2020. |  |
| Data collection process | 9 | | Data were independently extracted by four authors using a standardized data extraction format that was developed according to the 2014 Joanna Briggs Institute Reviewers’ Manual[21]. The tool includes Authors, country, and study year, study design, sample size, prevalence of depression among medical students, tool used to measure the outcome, response rate, and risk of bias assessment score were included in the extraction. The data were extracted by two independent reviewers and any inconsistent data was cross-checked (**Additional File 2**). The disagreement between the reviewers was solved by a thorough discussion. |  |
| Data items | 10a | | Articles with clear outcomes about depression, and published from 2013 to 2023 were included in this particular systematic review and Meta-analysis. |  |
|  | 10b | | Articles with clear outcomes about depression and low risk of bias |  |
| Study risk of bias assessment | 11 | | There are a total of 31 articles assessed for methodological quality using 9 point score tool developed by JBI for observational prevalence studies. The outcome of the quality appraisal ranged from moderate to high methodological quality in which eleven studies[22-32] score 9 points, eleven studies[15, 33-42] scored 8 points, four studies[14, 43-45] scored 7 points and the remaining five studies[46-50] scored 6 points.( **Additional File 1**). |  |
| Effect measures | 12 | | The odds ratio (OR), Logor, and standard error of logOr or (SelogOR) were used presentation of results. |  |
| Synthesis methods | 13a | | The qualitative and quantitative synthesis was employed. |  |
|  | 13b | | The pooled effect-size with random model of analysis was employed. |  |
|  | 13c | | We have used the PRISMA flow chart, forest plot, and funnel plot to present and visually displayed the data. |  |
|  | 13d | | The standard chi-square and I-square statical tests were used. The variation between different studies characteristics such as the country where the primary article conducted and outcome ascertainment tool were investigated through subgroup analyses. This subgroup analyses could demonstrate the sources of heterogeneity and let the researcher for another remedy such as the use of meta-regression to treat this heterogeneity. The influence of individual articles on the overall pooled effect size estimate or the prevalence of depression was assessed by using the sensitivity analysis. The forest plot with 95% CI was used to present the overall pooled prevalence as well as the subgroup pooled prevalence of depression among medical students in Africa. |  |
|  | 13e | | A random -effects model was used for analysis. |  |
|  | 13f | | We have conducted a sensitivity analysis to identify whether there is an evidence of influencing effect of one study on the other. The output of leave-one-out sensitivity analysis through random-effects model revealed that there was no any individual study that influenced the overall pooled estimate of depression among medical students in this particular review. For each single study, the effect size indicated relates with the overall pooled effect size generated from meta-analysis omitted that particular study **(Fig. 5**). |  |
| Reporting bias assessment | 14 | | We have used the Begg’s and/or Egger’s test to detect publication bias. |  |
| Certainty assessment | 15 | | The forest plot with 95% CI was used to present the overall pooled prevalence as well as the subgroup pooled prevalence of depression among medical students in Africa. |  |
| **RESULTS** | | | |  |
| Study selection | 16a | | In this systematic review and meta-analysis study a total of 2697 articles related to the prevalence of depression in African Medical students were identified using electronic databases and searching engine websites. Among overall articles found 2074 were removed for being irrelevance and duplicated and the other pretty sizable articles were removed for not being ineligible (study design and Title difference) by automation tools and other reasons (333 vs. 20) respectively. The remaining 88 articles were eligible for screening. Of these screened 37 papers were excluded due to region of study or not conducted in Africa and target population difference (those articles conducted among undergraduate University students). With further screening 51 articles were sought for retrieval and 9 were not retrieved by one and the other reason. Moreover, 42 research articles were assessed for eligibility to be included for the review process, but with the outcome of interest and measurement tool ambiguity a total of 10 articles were excluded. Finally, 31 original research articles were incorporated in the final systematic review and met-analysis (**Figure 1**). |  |
|  | 16b | | After a thorough examination of the titles and abstract using eligibility criteria, irrelevant studies were excluded. Then those articles considered relevant were reviewed; whereas those without clear reporting of the outcome of interest were excluded. |  |
| Study characteristics | 17 | | In this systematic review and meta-analysis, 34,189 participants were included with a response rate of 100%. The studies included in this review were observational cross-sectional studies published from 2016 to 2023. The smallest sample size was 92 from the study conducted in Morocco[39] followed by the studies 170 in Libya[48] and 203 in Morocco [34]. On the other hand, the largest sample size was 1058 from Sudan[42] followed by 1300 from Libya[35]. The prevalence of depression among medical students ranges from 10.10% to 75% from the studies in Nigeria and Sudan respectively [28, 42]. The articles were utilized five different types of outcome ascertainment tools such as HADS, BID, SRQ-20, PHQ-9, and DASS-21. In this regard 8(25.6%) studies used PHQ measuring tool, 7(22.6%) studies used BID and DASS-21 measuring tool to ascertain depression among medical students. Regarding risk of bias assessment we have used by JBI guidelines for observational prevalence studies with 9 point score. The quality score range was 6-9 among the included studies for this systematic review and meta-analysis (Table 1). . |  |
| Risk of bias in studies | 18 | | There are a total of 31 articles assessed for methodological quality using 9 point score tool developed by JBI for observational prevalence studies. For the nutshell, all articles had had high quality and included in the final analysis process. |  |
| Results of individual studies | 19 | | \| Author(Publication year) \| Country \| Tool used \| study design \| Population \| sample \| Prevalence \| Response rate \| Quality \| \| --- \| --- \| --- \| --- \| --- \| --- \| --- \| --- \| --- \| \| Kebede et al., 2019 [15] \| Ethiopia \| HADS \| Cross-sectional \| Medical students \| 273 \| 51.30 \| 98.5% \| 8 \| \| Dagnew et al., 2020 [33] \| Ethiopia \| BDI \| Cross-sectional \| Medical students \| 383 \| 34.73 \| 97.7% \| 8 \| \| S van der Walt et al., 2020 [22] \| South Africa \| HADS \| Cross-sectional \| Medical students \| 473 \| 25.00 \| 100% \| 9 \| \| Bawo O. James et al.,2017 [23] \| Nigeria \| HADS \| Cross-sectional \| Medical students \| 623 \| 21.30 \| 98.1% \| 9 \| \| Joshua Falade et al. , 2020 [24] \| Nigeria \| HADS \| Cross-sectional \| Medical students \| 944 \| 14.30 \| 97.8% \| 9 \| \| M. Barrimi et al. 2020 [47] \| Morocco \| BDI \| Cross-sectional \| Medical students \| 605 \| 10.40 \| 100% \| 6 \| \| Mboya et al. , 2020 [34] \| Tanzania \| SRQ-20 \| Cross-sectional \| Medical students \| 203 \| 14.3 \| 100% \| 8 \| \| Olum et al., 2020 [43] \| Uganda \| PHQ-9 \| Cross-sectional \| Medical students \| 331 \| 21.50 \| 93.8% \| 7 \| \| Ngasa et al., 2017 [25] \| Cameroon \| PHQ-9 \| Cross-sectional \| Medical students \| 618 \| 30.60 \| 90.4% \| 9 \| \| Njim T, et al., 2019 [36] \| Cameroon \| PHQ-9 \| Cross-sectional \| Medical students \| 413 \| 66.34 \| 82.6% \| 8 \| \| Edmund Ndudi Ossai et al., 2021 [31] \| Nigeria \| BDI \| Cross-sectional \| Medical students \| 522 \| 26.6 \| 100% \| 9 \| \| El-Gilany et al., 2019 [27] \| Egypt \| BDI \| Cross-sectional \| Medical students \| 900 \| 25.20 \| 100% \| 9 \| \| C. E. NWACHUKWU ET AL., 2021 [28] \| Nigeria \| HADS \| Cross-sectional \| Medical students \| 690 \| 10.10 \| 100% \| 9 \| \| Mohamed Fawzy et al., 20117 [14]. \| Egypt \| DASS-21 \| Cross-sectional \| Medical students \| 700 \| 65.00 \| 100% \| 7 \| \| Narushni Pillay et al., 2016 [46] \| South Africa \| DASS-21 \| Cross-sectional \| Medical students \| 230 \| 15.6 \| Not stated \| 6 \| \| Uzoechi Eze Chikezie et al., 2021 [44] \| Nigeria \| DASS-21 \| Cross-sectional \| Medical students \| 243 \| 25.5 \| 100% \| 7 \| \| Wafaa et al., 2020 [29] \| Egypt \| DASS-21 \| Cross-sectional \| Medical students \| 390 \| 45.1 \| 100% \| 9 \| \| Sherif RF et al., 2021 [48] \| Libya \| PHQ-9 \| Cross-sectional \| Medical students \| 170 \| 45 \| 100% \| 6 \| \| Suraj, et al., 2021 [37] \| Nigeria \| SRQ-20 \| Cross-sectional \| Medical students \| 279 \| 15.1 \| 100% \| 8 \| \| Leta Melaku et al., 2021 [30] \| Ethiopia \| DASS-21 \| Cross-sectional \| Medical students \| 260 \| 53 \| 98.1% \| 9 \| \| Tarteel Musa et al., 2022 [49] \| Sudan \| HADS \| Cross-sectional \| Medical students \| 355 \| 78 \| 100% \| 6 \| \| Khalid A. Khalil et al. [35] \| Libya \| PHQ-9 \| Cross-sectional \| Medical students \| 1300 \| 45 \| 74.6% \| 8 \| \| H Essangri et al., 2021[38] \| Morocco \| BDI \| Cross-sectional \| Medical students \| 549 \| 74.7 \| 100% \| 8 \| \| Rammouz et al., 2023 [39] \| Morocco \| BDI \| Cross-sectional \| Medical students \| 92 \| 41.3 \| 91.4% \| 8 \| \| Shereen Esmat et al., 2021 [40] \| Egypt \| BDI \| Cross-sectional \| Medical students \| 238 \| 38.2 \| 79.3% \| 8 \| \| Mwita M et al., 2020 [41] \| Tanzania \| PHQ-9 \| Cross-sectional \| Medical students \| 353 \| 41.36 \| 100% \| 8 \| \| Sserunkuuma, J., et al., 2023 [32] \| Uganda \| PHQ-9 \| Cross-sectional \| Medical students \| 269 \| 16.73 \| 100% \| 9 \| \| S. H. Mustafa et al., 2022 [51] \| Sudan \| SRQ-20 \| Cross-sectional \| Medical students \| 432 \| 55.8 \| 100% \| 9 \| \| Mohamed, E.A.A.,et al., 2018 [45] \| Sudan \| PHQ-9 \| Cross-sectional \| Medical students \| 440 \| 67 \| 100% \| 7 \| \| Dafaalla, M., et al., 2016 [50] \| Sudan \| DASS-21 \| Cross-sectional \| Medical students \| 487 \| 53.4 \| 97.4% \| 6 \| \| Nubi et al. ,2022 [42] \| Sudan \| DASS-21 \| Cross-sectional \| Medical students \| 1058 \| 75 \| 99.9% \| 8 \| |  |
| Results of syntheses | 20a | | We have assessed the risk of bias using Begg’s and/or Egger’s test. |  |
|  | 20b | | A total of 31 primary articles were appraised and retrieved to pool the prevalence of depression among medical students in Africa. The prevalence ranged from 10.10% to 75.0% from Nigeria and Sudan respectively [28, 42]. The pooled prevalence of depression among medical students was 38.80% [95%CI (29.55, 48.05), I^2^=100.00%, P<0.001]. The effect size of overall pooled prevalence of depression among medical students was presented using forest plot (**Fig.2).** |  |
|  | 20c | | In this systematic review and meta-analysis, the analysis output using random-effects model showed a high variability across the primary articles included in the study(I^2^= 100%, P<0.001). This variability is inevitable in meta-analysis studies resulting from quality difference of the included studies, methodological differences, sample size, inclusion and exclusion, and the difference in measuring tool to ascertain the outcome of interest. Therefore, we have conducted the meta-regression analysis by using publication year, sample size, and standard error as a covariates to figure out the potential source of heterogeneity among included studies. |  |
|  | 20d | | We have conducted a sensitivity analysis to identify whether there is an evidence of influencing effect of one study on the other. The output of leave-one-out sensitivity analysis through random-effects model revealed that there was no any individual study that influenced the overall pooled estimate of depression among medical students in this particular review. For each single study, the effect size indicated relates with the overall pooled effect size generated from meta-analysis omitted that particular study **(Fig. 5**). |  |
| Reporting biases | 21 | | In this regard, the meta-regression analysis revealed that no significant correlation found between the outcome of interest (depression) and the included covariates by far (p =0.662 for publication year and P= 0.686 for sample size). Hence, there was no statically significant association possible existence of variability as shown (Table 2). This again implies that the source of high variability (heterogeneity) could be due to chance or the other variables not investigated in this particular review. |  |
| Certainty of evidence | 22 | | In this systematic review and meta-analysis, the pooled prevalence of depression among medical students was 38.80% [95%CI (29.55, 48.05), I^2^=100.00%, P<0.001]. The effect size of overall pooled prevalence of depression among medical students was presented using forest plot (**Fig.2).** |  |
| **DISCUSSION** | | | |  |
| Discussion | 23a | | **Discussion**  This systematic review and meta-analysis was conducted to determine the pooled prevalence of depression and it’s associated among medical students in Africa. The overall pooled prevalence of depression among African medical students was 38.80% [95%CI (29.55, 48.05), I^2^=100.00%, P<0.001]. The current meta-analysis result is consistent with the studies conducted in China [53] and India[54] with pooled prevalence of 37.9% and 40% of medical students experienced depression respectively. But, it was higher than the studies conducted in China and Spain [55-57] with the pooled prevalence of 27%, 29%, and 31% respectively. The possible explanation for the variation between the current study and Spain was time gap and sample size difference in which the review in Spain was conducted only from articles published from 2019 to 2020 during the COVID-19 pandemic. Moreover, the current is higher than the study conducted in China with a pooled prevalence of 33.7% of the medical students experienced depression [58]. The possible discrepancy might be due to the fact that there are socio-economic, sociocultural, number of reviewed article, tool and time variations with the current systematic review and meta-analysis to that of China. There is also time gap which accounts the variation in the proportion of depression across the Nations.  It was also higher than the studies conducted in Singapore [59] 28.0%, and Brazil [60, 61] 30.6% and 28.51% pooled prevalence of depression among medical students. Moreover, the finding of this systematic review and meta-analysis is higher than the studies conducted in America with 27.2% pooled prevalence of depression [62], China [63] with the pooled prevalence of 27% among medical students. The possible variation might be due to the fact that there is population difference in Assia and Africa. Furthermore; the current study finding is higher than the study conducted in China [64] with a pooled prevalence of 19.9%. The difference might be due to the study in China conducted only in one nation University Medical Students whereas the current study is over African Universities which might cause the discrepancy.  However, the current finding is lower than the study conducted in North America 66.5% of medical students experienced depression [66]. The possible explanation might be the review in North America included all articles published between the year 1906 to 2013 which causes sample variation, tool variation, and time gap. The finding was lower than the reviews conducted in China [67] and India[65] with the pooled prevalence of depression ranges from 13.10 to 76.21% and 50.% respectively. The possible explanation for the variation might be due sociocultural variation, sample size difference and the review in India included only studies published between 2019 to 2020 that was during COVID-19 pandemic which probably increase the depression among medical students due to lockdown, long time social isolation, and perceived probability of encountering the pandemic. This had been supported by the study conducted in Bahraine [66]. Besides, the current systematic review and meta-analysis was conducted in Africa which is a continent with 56 countries, but the above reviews conducted in individual countries which could a reason for discrepancy. Therefore; further global based studies to assess other factors related to depression ought to be done. |  |
|  | 23b | | However this review has its limitations such as it included only articles published in English were included in this systematic review and meta-analysis**.** The primary studies were all observational cross-sectional studies**.** In addition, the meta-analysis didn’t include the factors attributed for depression among medical students, and only included the studies conducted in Africa. |  |
|  | 23c | | In addition, the meta-analysis didn’t include the factors attributed for depression among medical students, and only included the studies conducted in Africa. It didn’t address all the global evidence published elsewhere with any language. |  |
|  | 23d | | The output of this systematic review and meta-analysis provides an up-to-date body of knowledge about the magnitude and severity of depression among medical students in Africa which is an alarming aggregated and rigorous evidence to think about and devise strategies to tackle it. Moreover, the finding has had an important clinical implication in providing pooled evidence of depression for those who are interested in the area, particularly for those focusing on mental health and wellbeing. Furthermore, this study has its own strength in generating pooled data in Africa which could help those in the need of such an evidence to devise strategies to tackle depression. |  |
| **OTHER INFORMATION** | | | |  |
| Registration and protocol | 24a | This systematic review and meta-analysis title and its protocol were registered in the PROSPERO online database (with registration number CRD42023431163). | |  |
|  | 24b | This particular review protocol can be accessed via online databases. | |  |
|  | 24c | The amendments for this review might be done /might not be done. | |  |
| Support | 25 | All the Authors did not receive any fund for to accomplish this review study so far. | |  |
| Competing interests | 26 | The authors have declared that there are no competing interests. | |  |
| Availability of data, code and other materials | 27 | The data extracted for this review was analysed and reported in the main document. | |  |

*From:*  Page MJ, McKenzie JE, Bossuyt PM, Boutron I, Hoffmann TC, Mulrow CD, et al. The PRISMA 2020 statement: an updated guideline for reporting systematic reviews. BMJ 2021;372:n71. doi: 10.1136/bmj.n71

For more information, visit: <http://www.prisma-statement.org/>
