# Supplementary material for: Prevalence of depression among medical students in Africa: Systematic review and meta-analysis
Source: PLoS One. 2024 Dec 26;19(12):e0312281. doi: 10.1371/journal.pone.0312281 (PMC11670985; doi:10.1371/journal.pone.0312281)
Supplement: S2 File — (DOCX) [file pone.0312281.s002.docx]

| **ID** | **Publication year** | **Author/s** | **Tool used** | **Country** | **Study design** | **sample size** | **r(pwo)^*^** | **logp** | **prevalence** | **Response rate** | **oddsp** | **logoddsp** | **sep** | **Selogoddsp** |
| --- | --- | --- | --- | --- | --- | --- | --- | --- | --- | --- | --- | --- | --- | --- |
| 1 | 2019 | Kebede et al. | HADS | Ethiopia | Crossectional | 273 | 140 | 1.71 | 51.30 | 98.50 | 1.05 | 0.05 | 1.28 | 0.10 |
| 2 | 2020 | Dagnew et al. | BDI | Ethiopia | Crossectional | 383 | 133 | 1.54 | 34.73 | 97.70 | 0.47 | -0.63 | 1.00 | 0.10 |
| 3 | 2020 | S van der Walt et al. | HADS | South Africa | Crossectional | 473 | 118 | 1.40 | 25.00 | 100.00 | 0.46 | -1.10 | 0.79 | 0.10 |
| 4 | 2017 | Bawo O. James et al. | HADS | Nigeria | Crossectional | 623 | 133 | 1.33 | 21.30 | 98.10 | 0.39 | -1.31 | 0.58 | 0.10 |
| 5 | 2020 | Joshua Falade et al. | HADS | Nigeria | Crossectional | 944 | 135 | 1.16 | 14.30 | 97.80 | 0.17 | -1.79 | 0.34 | 0.09 |
| 6 | 2020 | M. Barrimi et al. | BDI | Morocco | Crossectional | 605 | 63 | 1.02 | 10.40 | 100.00 | 0.22 | -2.15 | 0.48 | 0.13 |
| 7 | 2020 | Mboya et al. | SRQ-20 | Tanzania | Crossectional | 203 | 29 | 1.16 | 14.30 | 100.00 | 0.17 | -1.79 | 2.46 | 0.20 |
| 8 | 2020 | Olum et al | PHQ-9 | Uganda | Crossectional | 331 | 71 | 1.33 | 21.50 | 94.00 | 0.27 | -1.30 | 1.10 | 0.13 |
| 9 | 2017 | Ngasa et al | PHQ-9 | Cameroon | Crossectional | 618 | 190 | 1.49 | 30.60 | 90.00 | 0.56 | -0.82 | 0.62 | 0.08 |
| 10 | 2018 | Njim T, et al | PHQ-9 | Cameroon | Crossectional | 413 | 274 | 1.82 | 66.34 | 82.60 | 2.62 | 0.68 | 0.66 | 0.08 |
| 11 | 2021 | Edmund Ndudi Ossai et al | BDI | Nigeria | Crossectional | 522 | 91 | 1.42 | 26.60 | 100.00 | 0.36 | -1.02 | 0.73 | 0.11 |
| 12 | 2019 | El-Gilany et al | BDI | Egypt | Crossectional | 900 | 227 | 1.40 | 25.20 | 100.00 | 0.41 | -1.09 | 0.42 | 0.07 |
| 13 | 2021 | C. E. NWACHUKWU ET AL. | HADS | Nigeria | Crossectional | 690 | 70 | 1.00 | 10.10 | 100.00 | 0.17 | -2.19 | 0.41 | 0.13 |
| 14 | 2017 | Mohamed Fawzy | DASS-21 | Egypt | Crossectional | 700 | 455 | 1.81 | 65.00 | 100.00 | 1.86 | 0.62 | 0.40 | 0.06 |
| 15 | 2016 | Narushni Pillay et al. | DASS-21 | South Africa | Crossectional | 230 | 35 | 1.19 | 15.60 | NA | 0.19 | -1.69 | 1.45 | 0.18 |
| 16 | 2021 | Uzoechi Eze Chikezie et al. | DASS-21 | Nigeria | Crossectional | 243 | 62 | 1.41 | 25.5 | 100.00 | 0.77 | -1.07 | 1.55 | 0.14 |
| 17 | 2020 | Wafaa et al. | DASS-21 | Egypt | Crossectional | 390 | 176 | 1.65 | 45.1 | 100.00 | 0.97 | -0.20 | 0.95 | 0.09 |
| 18 | 2021 | Sherif RF et al. | PHQ-9 | Lybia | Crossectional | 170 | 76 | 1.65 | 45 | 100.00 | 1.80 | -0.20 | 2.17 | 0.14 |
| 19 | 2021 | Suraj, et al. | SRQ-20 | Nigeria | Crossectional | 279 | 42 | 1.18 | 15.1 | 100.00 | 0.15 | -1.73 | 1.18 | 0.17 |
| 20 | 2021 | Leta Melaku et al. | DASS-21 | Ethiopia | Crossectional | 260 | 138 | 1.72 | 53 | 98.10 | 0.53 | 0.12 | 1.32 | 0.11 |
| 21 | 2022 | Tarteel Musa et al. | HADS | Sudan | Crossectional | 355 | 277 | 1.89 | 78 | 100.00 | 0.78 | 1.27 | 0.55 | 0.08 |
| 22 | 2019 | Khalid A. Khalil | PHQ-9 | Lybia | Crossectional | 1300 | 585 | 1.65 | 45 | 74.60 | 0.45 | -0.20 | 0.28 | 0.05 |
| 23 | 2021 | H Essangri et al. | BDI | Morocco | Crossectional | 549 | 410 | 1.87 | 74.7 | 100.00 | 0.75 | 1.08 | 0.40 | 0.07 |
| 24 | 2023 | Rammouz et al. | BDI | Morocco | Crossectional | 92 | 38 | 1.62 | 41.3 | 91.40 | 0.41 | -0.35 | 4.10 | 0.19 |
| 25 | 2021 | Shereen Esmat et al. | BDI | Egypt | Crossectional | 238 | 91 | 1.58 | 38.2 | 79.30 | 0.38 | -0.48 | 1.60 | 0.12 |
| 26 | 2020 | Mwita M et al. | PHQ-9 | Tanzania | Crossectional | 353 | 146 | 1.62 | 41.36 | 100.00 | 0.41 | -0.35 | 1.07 | 0.10 |
| 27 | 2023 | Sserunkuuma, J., et al | PHQ-9 | Uganda | Crossectional | 269 | 45 | 1.22 | 16.73 | 100.00 | 0.17 | -1.60 | 1.27 | 0.16 |
| 28 | 2020 | S. H. Mustafa et al. | SRQ-20 | Sudan | Crossectional | 432 | 241 | 1.75 | 55.8 | 100.00 | 0.56 | 0.23 | 0.76 | 0.08 |
| 29 | 2018 | Mohamed, E.A.A.,et al. | PHQ-9 | Sudan | Crossectional | 440 | 295 | 1.83 | 67 | 100.00 | 0.67 | 0.71 | 0.61 | 0.08 |
| 30 | 2016 | Dafaalla, M., et al. | DASS-21 | Sudan | Crossectional | 487 | 260 | 1.73 | 53.4 | 97.40 | 0.53 | 0.14 | 0.70 | 0.08 |
| 31 | 2022 | Nubi et al. | DASS-21 | Sudan | Crossectional | 1058 | 794 | 1.88 | 75 | 99.90 | 0.75 | 1.10 | 0.20 | 0.05 |

**S2 File**: Data extraction

Note: **r(pwo)=people with outcome, NA= Not Applicable**

**Factors associated with depression**

Being Female medical student as a factor for depression

| **ID** | **publication year** | **Author/s** | **Tool used** | **Country** | **study design** | **sample size** | **r(pwo)** | **logp** | **p** | sample | AOR | UBCI | LBCI | logor | selogor |
| --- | --- | --- | --- | --- | --- | --- | --- | --- | --- | --- | --- | --- | --- | --- | --- |
| 1 | 2019 | Kebede et al. | HADS | Ethiopia | Crossectional | 273 | 140 | 1.710117365 | 51.30 | 273 | 0.99 | 1.75 | 0.57 | -0.01005 | 1.604592 |
| 2 | 2020 | S van der Walt et al. | HADS | South Africa | Crossectional | 473 | 118 | 1.397940009 | 25.00 | 473 | 3.7 | 6.8 | 2 | 1.308333 | 6.289796 |
| 3 | 2020 | M. Barrimi et al. |  | Morocco | Crossectional | 605 | 63 | 1.017033339 | 10.40 | 605 | 0.16 | 0.33 | 0.07 | -1.83258 | 0.312143 |
| 4 | 2020 | Olum et al | PHQ9 score | Uganda | Crossectional | 331 | 71 | 1.33243846 | 21.50 | 331 | 1.11 | 2 | 0.59 | 0.10436 | 1.84949 |
| 5 | 2017 | Ngasa et al | PHQ-9 score | Cameroon | Crossectional | 618 | 190 | 1.485721426 | 30.60 | 618 | 1.59 | 2.37 | 1.06 | 0.463734 | 2.099592 |
| 6 | 2019 | El-Gilany et al | Beck´s Depression Inventory (BDI) | Egypt | Crossectional | 900 | 227 | 1.401400541 | 25.20 | 900 | 0.59 | 0.91 | 0.38 | -0.52763 | 0.813061 |
| 7 | 2021 | C. E. NWACHUKWU ET AL. |  | Nigeria | Crossectional | 690 | 70 | 1.004321374 | 10.10 | 690 | 1.2 | 1.89 | 0.79 | 0.182322 | 1.688469 |
| 8 | 2020 | Wafaa et al. | DASS 21 | Egypt | Crossectional | 390 | 176 | 1.654176542 | 45.1 | 390 | 1.7 | 2.71 | 1.04 | 0.530628 | 2.444694 |
| 9 | 2021 | Suraj, et al. |  | Nigeria | Crossectional | 279 | 42 | 1.178976947 | 15.1 | 279 | 2.88 | 6.33 | 1.21 | 1.05779 | 6.021327 |
| 10 | 2021 | Leta Melaku et al. | Depression, Anxiety, Stress Scale(DASS-21) | Ethiopia | Crossectional | 260 | 138 | 1.72427587 | 53 | 260 | 1.33 | 2.94 | 1.05 | 0.285179 | 2.672143 |
| 11 | 2021 | Shereen Esmat et al. | Beck’s Depression Inventory | Egypt | Crossectional | 238 | 91 | 1.582063363 | 38.2 | 238 | 2.44 | 5.21 | 1.14 | 0.891998 | 4.919184 |
| 12 | 2020 | Mwita M et al. | (PHQ-9) | Tanzania | Crossectional | 353 | 146 | 1.61658053 | 41.36 | 353 | 1.43 | 2 | 0.83 | 0.357674 | 1.788265 |

**Year of study (first, second, third, fourth, fifth and sixth)**

| **ID** | **publication year** | **Author/s** | **Tool used** | **Country** | **study design** | **sample size** | **r(pwo)** | **logp** | **p** | **AOR** | **UBCI** | **LBCI** | **LogOR** | **selogOR** |
| --- | --- | --- | --- | --- | --- | --- | --- | --- | --- | --- | --- | --- | --- | --- |
| 1 | 2019 | Kebede et al. | HADS | Ethiopia | Crossectional | 273 | 140 | 1.710117 | 51.30 | 1.63 | 6.26 | 1.43 | 0.48858 | 5.895204 |
| 2 | 2020 | Olum et al | PHQ-9 | Uganda | Crossectional | 331 | 71 | 1.332438 | 21.50 | 1.43 | 5 | 0.5 | 0.357674 | 4.872449 |
| 3 | 2021 | Leta Melaku et al. | DASS-21 | Ethiopia | Crossectional | 260 | 138 | 1.724276 | 53 | 1.91 | 4.28 | 0.85 | 0.647103 | 4.063163 |
| 4 | 2020 | Mwita M et al. | PHQ-9 | Tanzania | Crossectional | 353 | 146 | 1.616581 | 41.36 | 1.43 | 5 | 0.53 | 0.357674 | 4.864796 |
| 5 | 2021 | Suraj, et al. | SRQ-20 | Nigeria | Crossectional | 279 | 42 | 1.178977 | 15.1 | 2.88 | 4.67 | 1.72 | 1.05779 | 4.231224 |

**Second year**

| **ID** | **publication year** | **Author/s** | **Tool used** | **Country** | **study design** | **sample size** | **r(pwo)** | **logp** | **p** | **logoddsp** | **sep** | **Selogoddsp** | **AOR** | **UBCI** | **LBCI** | **LogOR** | **seLogOR** |
| --- | --- | --- | --- | --- | --- | --- | --- | --- | --- | --- | --- | --- | --- | --- | --- | --- | --- |
| 1 | 2019 | Kebede et al. | HADS | Ethiopia | Crossectional | 273 | 140 | 1.710117 | 51.30 | 0.05 | 1.27768853 | 0.104015997 | 1.39 | 5.18 | 1.57 | 0.329304 | 4.7794898 |
| 2 | 2020 | Olum et al | PHQ-9 | Uganda | Crossectional | 331 | 71 | 1.332438 | 21.50 | -1.30 | 1.09966624 | 0.130823584 | 1.6 | 3.8 | 0.7 | 0.470004 | 3.62142857 |
| 3 | 2021 | Leta Melaku et al. | DASS-21 | Ethiopia | Crossectional | 260 | 138 | 1.724276 | 53 | 0.120144312 | 1.31601986 | 0.105391559 | 4.5 | 10.73 | 1.88 | 1.504077 | 10.2504082 |
| 4 | 2020 | Mwita M et al. | PHQ-9 | Tanzania | Crossectional | 353 | 146 | 1.616581 | 41.36 | -0.349102827 | 1.06834008 | 0.098438936 | 0.24 | 0.53 | 0.11 | -1.42712 | 0.50193878 |
| 5 | 2021 | Suraj, et al. | SRQ-20 | Nigeria | Crossectional | 279 | 42 | 1.178977 | 15.1 | 1.182474981 | 0.16554953 | 0.165549528 | 5.11 | 9.46 | 3.23 | 1.631199 | 8.63602041 |

**Third year**

| **ID** | **publication year** | **Author/s** | **Tool used** | **Country** | **study design** | **sample size** | **r(pwo)** | **logp** | **p** | **logoddsp** | **sep** | **Selogoddsp** | **AOR** | **UBCI** | **LBCI** | **LogOR** | **seLogOR** |
| --- | --- | --- | --- | --- | --- | --- | --- | --- | --- | --- | --- | --- | --- | --- | --- | --- | --- |
| 1 | 2019 | Kebede et al. | HADS | Ethiopia | Crossectional | 273 | 140 | 1.710117 | 51.30 | 0.05 | 1.277689 | 0.104015997 | 0.5 | 1.65 | 0.16 | -0.69315 | 1.60918367 |
| 2 | 2020 | Olum et al | PHQ-9 | Uganda | Crossectional | 331 | 71 | 1.332438 | 21.50 | -1.30 | 1.099666 | 0.130823584 | 1.3 | 3.6 | 0.5 | 0.262364 | 3.47244898 |
| 3 | 2021 | Leta Melaku et al. | DASS-21 | Ethiopia | Crossectional | 260 | 138 | 1.724276 | 53 | 0.120144 | 1.31602 | 0.105391559 | 4.85 | 12.19 | 1.93 | 1.578979 | 11.6976531 |
| 4 | 2020 | Mwita M et al. | PHQ-9 | Tanzania | Crossectional | 353 | 146 | 1.616581 | 41.36 | -0.3491 | 1.06834 | 0.098438936 | 0.36 | 0.77 | 0.16 | -1.02165 | 0.72918367 |
| 5 | 2021 | Suraj, et al. | SRQ-20 | Nigeria | Crossectional | 279 | 42 | 1.178977 | 15.1 | 1.182475 | 0.16555 | 0.165549528 | 9.37 | 14.78 | 3.89 | 2.237513 | 13.7876531 |

Fourth year

| **ID** | **publication year** | **Author/s** | **Tool used** | **Country** | **study design** | **sample size** | **r(pwo)** | **logp** | **p** | **logoddsp** | **sep** | **Selogoddsp** | **AOR** | **UBCI** | **LBCI** | **LogOr** | **SeLogOr** |
| --- | --- | --- | --- | --- | --- | --- | --- | --- | --- | --- | --- | --- | --- | --- | --- | --- | --- |
| 1 | 2019 | Kebede et al. | HADS | Ethiopia | Crossectional | 273 | 140 | 1.710117 | 51.30 | 0.05 | 1.2777 | 0.104016 | 0.52 | 1.68 | 0.16 | -0.653926467 | 1.4896 |
| 2 | 2020 | Olum et al | PHQ-9 | Uganda | Crossectional | 331 | 71 | 1.332438 | 21.50 | -1.30 | 1.0997 | 0.130824 | 0.4 | 1.2 | 0.1 | -0.916290732 | 1.078 |
| 3 | 2021 | Leta Melaku et al. | DASS-21 | Ethiopia | Crossectional | 260 | 138 | 1.724276 | 53 | 0.120144 | 1.316 | 0.105392 | 2.34 | 5.22 | 1.05 | 0.850150929 | 4.0866 |
| 4 | 2020 | Mwita M et al. | PHQ-9 | Tanzania | Crossectional | 353 | 146 | 1.616581 | 41.36 | -0.3491 | 1.0683 | 0.098439 | 0.56 | 1.43 | 0.23 | -0.579818495 | 1.176 |
| 5 | 2021 | Suraj, et al. | SRQ-20 | Nigeria | Crossectional | 279 | 42 | 1.178977 | 15.1 | 1.182475 | 0.1655 | 0.16555 | 8.52 | 16.32 | 2.96 | 2.142416341 | 13.0928 |

Fifth year

| **ID** | **publication year** | **Author/s** | **Tool used** | **Country** | **study design** | **sample size** | **r(pwo)** | **p** | **AOR** | **UBCI** | **LBCI** | **logOR** | **SelogOR** |
| --- | --- | --- | --- | --- | --- | --- | --- | --- | --- | --- | --- | --- | --- |
| 1 | 2019 | Kebede et al. | HADS | Ethiopia | Crossectional | 273 | 140 | 51.30 | 1.9 | 8.81 | 0.41 | 0.641853886 | 2.142857143 |
| 2 | 2020 | Olum et al | PHQ-9 | Uganda | Crossectional | 331 | 71 | 21.50 | 0.7 | 2 | 0.2 | -0.356674944 | 0.459183673 |
| 3 | 2021 | Leta Melaku et al. | DASS-21 | Ethiopia | Crossectional | 260 | 138 | 53 | 2.01 | 4.94 | 0.82 | 0.698134722 | 1.051020408 |
| 4 | 2020 | Mwita M et al. | PHQ-9 | Tanzania | Crossectional | 353 | 146 | 41.36 | 0.7 | 1.9 | 0.2 | -0.356674944 | 0.433673469 |
| 5 | 2021 | Suraj, et al. | SRQ-20 | Nigeria | Crossectional | 279 | 42 | 15.1 | 0.66 | 2.87 | 0.13 | -0.415515444 | 0.698979592 |

Clinical stage of students(preclinical and clinical)

| **ID** | **publication year** | **Author/s** | **Tool used** | **Country** | **study design** | **sample size** | **r(pwo)** | **logp** | **p** | **logoddsp** | **sep** | **Selogoddsp** | **AOR** | **UBCI** | **LBCI** | **LogOr** | **SelogOr** |
| --- | --- | --- | --- | --- | --- | --- | --- | --- | --- | --- | --- | --- | --- | --- | --- | --- | --- |
| 1 | 2020 | S van der Walt et al. | HADS | South Africa | Crossectional | 473 | 118 | 1.39794 | 25.00 | -1.10 | 0.792811839 | 0.102923371 | 1.2 | 2 | 0.7 | 0.182321557 | 1.821428571 |
| 2 | 2017 | Ngasa et al | PHQ-9 | Cameroon | Crossectional | 618 | 190 | 1.485721 | 30.60 | -0.82 | 0.621200374 | 0.082969292 | 4.26 | 6.71 | 2.71 | 1.44926916 | 6.018673469 |
| 3 | 2019 | El-Gilany et al | BDI | Egypt | Crossectional | 900 | 227 | 1.401401 | 25.20 | -1.09 | 0.417214467 | 0.074280774 | 0.67 | 0.91 | 0.46 | -0.400477567 | 0.792653061 |
| 4 | 2021 | C. E. NWACHUKWU ET AL. | HADS | Nigeria | Crossectional | 690 | 70 | 1.004321 | 10.10 | -2.19 | 0.414067637 | 0.125447571 | 4.1 | 7.25 | 2.36 | 1.410986974 | 6.647959184 |

Social support of medical students

| **ID** | **publication year** | **Author/s** | **Tool used** | **Country** | **study design** | **sample size** | **r(pwo)** | **logp** | **p** | **logoddsp** | **sep** | **Selogoddsp** | **AOR** | **UBCI** | **LBCI** | **LogOr** | **SeLogOr** |  |
| --- | --- | --- | --- | --- | --- | --- | --- | --- | --- | --- | --- | --- | --- | --- | --- | --- | --- | --- |
| 1 | 2019 | Kebede et al. | HADS | Ethiopia | Crossectional | 273 | 140 | 1.710117 | 51.30 | 0.05 | 1.277689 | 0.104016 | 1.32 | 2.73 | 0.63 | 0.277632 | 2.502959 |  |
| 2 | 2021 | Suraj, et al. | SRQ-20 | Nigeria | Crossectional | 279 | 42 | 1.178977 | 15.1 | 1.182475 | 0.16555 | 0.16555 | 1.46 | 2.39 | 0.89 | 0.378436 | 1.823673 |  |
| 3 | 2020 | Mboya et al. | SRQ-20 | Tanzania | Crossectional | 402 | 56 | 1.143015 | 13.9 | 0.16144 | -1.82362 | 1.725424 | 4.55 | 9.1 | 2.22 | 1.515127 | 9.1 |  |
