# Supplementary material for: Prevalence of depression among medical students in Africa: Systematic review and meta-analysis
Source: PLoS One. 2024 Dec 26;19(12):e0312281. doi: 10.1371/journal.pone.0312281 (PMC11670985; doi:10.1371/journal.pone.0312281)
Supplement: S3 File — (DOCX) [file pone.0312281.s003.docx]

**S3 File**: **The methodological quality of 31 included studies for the prevalence of depression among medical students in Africa**

| Included articles | Q1 | Q2 | Q3 | Q4 | Q5 | Q6 | Q7 | Q8 | Q9 | Score/9 |
| --- | --- | --- | --- | --- | --- | --- | --- | --- | --- | --- |
| Kebede et al. [15] | Y | Y | Y | N | Y | Y | Y | Y | Y | 8 |
| Dagnew et al. [33]. | Y | Y | Y | Y | N | Y | Y | Y | Y | 8 |
| S van der Walt et al.[22] | Y | Y | Y | Y | Y | N | Y | Y | Y | 9 |
| Bawo O. James et al.[23] | Y | Y | Y | Y | Y | Y | Y | Y | Y | 9 |
| Joshua Falade et al.[24] | Y | Y | Y | Y | Y | Y | Y | Y | Y | 9 |
| M. Barrimi et al.[47] | Y | Y | N | UN | N | Y | Y | Y | Y | 6 |
| Mboya et al.[34] | Y | Y | N | Y | Y | Y | Y | Y | Y | 8 |
| Olum et al.[43] | Y | Y | Y | N | Y | Y | Y | Y | UN | 7 |
| Ngasa et al. [25] | Y | Y | Y | Y | Y | Y | Y | Y | Y | 9 |
| Njim T, et al.[36] | Y | Y | Y | Y | Y | Y | Y | Y | UN | 8 |
| Edmund Ndudi Ossai et al.[31] | Y | Y | Y | Y | Y | Y | Y | Y | Y | 9 |
| El-Gilany et al.[27] | Y | Y | Y | Y | Y | Y | Y | Y | Y | 9 |
| C. E. NWACHUKWU ET AL.[28] | Y | Y | Y | Y | Y | Y | Y | Y | Y | 9 |
| Mohamed Fawzy et al.[14] | Y | Y | Y | N | Y | N | Y | Y | Y | 7 |
| Narushni Pillay et al.[46] | Y | Y | N | Y | Y | Y | N | Y | UN | 6 |
| Uzoechi Eze Chikezie et al.[44] | Y | Y | Y | N | Y | Y | Y | N | Y | 7 |
| Wafaa et al.[29] | Y | Y | Y | Y | Y | Y | Y | Y | Y | 9 |
| Sherif RF et al.[48] | Y | N | Y | N | Y | Y | N | Y | Y | 6 |
| Suraj, et al.[37] | Y | Y | Y | Y | N | Y | Y | Y | Y | 8 |
| Leta Melaku et al.[30] | Y | Y | Y | Y | Y | Y | Y | Y | Y | 9 |
| Tarteel Musa et al.[49] | Y | Y | N | Y | N | Y | Y | Y | UN | 6 |
| Khalid A. Khalil et al. [35] | Y | Y | Y | Y | Y | Y | Y | Y | N | 8 |
| H Essangri et al.[38] | Y | Y | Y | N | Y | Y | Y | Y | Y | 8 |
| Rammouz et al.[39] | Y | Y | Y | N | Y | Y | Y | Y | Y | 8 |
| Shereen Esmat et al.[40] | Y | Y | Y | N | Y | Y | Y | Y | Y | 8 |
| Mwita M et al.[41] | Y | Y | Y | N | Y | Y | Y | Y | Y | 8 |
| Sserunkuuma, J., et al.[32] | Y | Y | Y | Y | Y | Y | Y | Y | Y | 9 |
| S. H. Mustafa et al.[51] | Y | Y | Y | Y | Y | Y | Y | Y | Y | 9 |
| Mohamed, E.A.A.,et al. [45] | UN | Y | Y | UN | Y | Y | Y | Y | Y | 7 |
| Dafaalla, M., et al. [50] | Y | N | Y | Y | Y | Y | N | Y | N | 6 |
| Nubi et al,.[42] | Y | Y | Y | N | Y | Y | Y | Y | Y | 8 |

N=No, NA= Not Applicable, UN= Unclear, Y=Yes,

Q1: Was the sample frame appropriate to address the target population? Q2: Were study participants sampled appropriately? Q3: Was the sample size adequate? Q4: Were the study subjects and the setting described in detail? Q5: Was the data analysis conducted with sufficient coverage of the identified sample? Q6: Were valid methods used for the identification of the condition? Q7: Was the condition measured in a standard, reliable way for all participants? Q8: Was there an appropriate statistical analysis? Q9: Was the response rate adequate and if not, was the low response rate managed appropriately?
