# Supplementary material for: Prevalence of depression among medical students in Africa: Systematic review and meta-analysis
Source: PLoS One. 2024 Dec 26;19(12):e0312281. doi: 10.1371/journal.pone.0312281 (PMC11670985; doi:10.1371/journal.pone.0312281)
Supplement: S1 Table — (DOCX) [file pone.0312281.s004.docx]

**S1 Table:** All studies identified in the literature search, including those that were excluded from the analyses.

| S/N | Author/s(reference | Title | DOI | Included OR Excluded | Reasons for exclusion | Published or unpublished | URL if unpublished |
| --- | --- | --- | --- | --- | --- | --- | --- |
| 1 | Kebede et al., 2019 [1] | *Prevalence and predictors of depression and anxiety among medical students in Addis Ababa, Ethiopia.* | <https://doi.org/10.1186/s13033-019-0287-6> | Included |  | Published |  |
| 2 | Dagnew et al., 2020 [2] | *Depression and Its Determinant Factors Among University of Gondar Medical and Health Science Students, Northwest Ethiopia: Institution-Based Cross-Sectional Study* | <https://doi.org/10.2147/ndt.s248409> | Included |  | Published |  |
| 3 | S van der Walt et al., 2020 [3] | *The burden of depression and anxiety among medical students in South Africa: A cross-sectional survey at the University of Cape Town* | <https://doi.org/10.7196/samj.2019.v110i1.14151> | Included |  | Published |  |
| 4 | Bawo O. James et al.,2017 [4] | *Psychosocial correlates of perceived stress among undergraduate medical students in Nigeria* | <https://doi.org/10.5116/ijme.59c6.3075> | Included |  | Published |  |
| 5 | Joshua Falade et al., 2020 [5] | *Prevalence and correlates of psychiatric morbidity, comorbid anxiety and depression among medical students in public and private tertiary institutions in a Nigerian state: a cross-sectional analytical study* | <https://doi.org/10.11604/pamj.2020.37.53.24994> | Included |  | Published |  |
| 6 | M. Barrimi et al. 2020 [6] | *Particularities of psychiatric and dermatological comorbidity among medical students in Morocco: Multicenter study* | <https://doi.org/10.1016/j.encep.2020.03.006> | Included |  | Published |  |
| 7 | Mboya et al. , 2020 [7] | *Factors associated with mental distress among undergraduate students in northern Tanzania* | <https://doi.org/10.1186/s12888-020-2448-1> | Included |  | Published |  |
| 8 | Olum et al., 2020 [8] | *Prevalence and factors associated with depression among medical students at Makerere University, Uganda.* | <https://doi.org/10.2147/amep.s278841> | Included |  | Published |  |
| 9 | Ngasa et al., 2017 [9] | *Prevalence and factors associated with depression among medical students in Cameroon: a cross-sectional study* | <https://doi.org/10.1186/s12888-017-1382-3> | Included |  | Published |  |
| 10 | Njim T, et al., 2019 [10] | *Burnout as a correlate of depression among medical students in Cameroon: a cross-sectional study* | <https://doi.org/10.1136/bmjopen-2018-027709> | Included |  | Published |  |
| 11 | Edmund Ndudi Ossai et al., 2021 [11] | *How large is the burden of depression in a medical school? A cross-sectional study among medical students in Nigeria* | <https://doi.org/10.11604/pamj.2021.40.71.29079> | Included |  | Published |  |
| 12 | El-Gilany et al., 2019 [12] | *Mental health status of medical students: a single faculty study in Egypt.* | <https://doi.org/10.1097/nmd.0000000000000970> | Included |  | Published |  |
| 13 | C. E. NWACHUKWU ET AL., 2021 [13] | *Common mental health problems and associated factors among medical students of University of Ibadan, Nigeria.* | <https://doi.org/10.1080/09638237.2021.1875404> | Included |  | Published |  |
| 14 | Mohamed Fawzy et al., 20117 [14]. | *Prevalence of psychological stress, depression and anxiety among medical students in Egypt.* | <https://doi.org/10.1016/j.psychres.2017.05.027> | Included |  | Published |  |
| 15 | Narushni Pillay et al., 2016 [15] | Burns, *Spirituality, depression and quality of life in medical students in KwaZulu-Natal.* | <https://doi.org/10.4102/sajpsychiatry.v22i1.731> | Included |  | Published |  |
| 16 | Uzoechi Eze Chikezie et al., 2021 [16] | *Prevalence of Depression and Associated Factors among Medical Students in a Southern Nigerian University.* | <https://doi.org/10.5539/gjhs.v13n12p12> | Included |  | Published |  |
| 17 | Wafaa et al., 2020 [17] | *Depression among medical students in Alexandria, Egypt.* | <https://doi.org/10.4314/ahs.v20i3.47> | Included |  | Published |  |
| 18 | Sherif RF et al., 2021 [18] | *Prevalence of depression among Libyan medical students* | <https://dx.doi.org/10.5281/zenodo.4723954> | Included |  | Published |  |
| 19 | Suraj, et al., 2021 [19] | *Prevalence and factors associated with depression among medical students in Nigeria* | <https://doi.org/10.4103/npmj.npmj_414_21> | Included |  | Published |  |
| 20 | Leta Melaku et al., 2021 [20] | *The Prevalence and Severity of Depression, Anxiety and Stress as Well as Coping Strategies Used by Medical Undergraduate Students Enrolled in Arsi University: A Cross-Sectional Study.* 2021. | <https://doi.org/10.21203/rs.3.rs-152511/v1> | Included |  | unpublished | https://www.researchsquare.com/article/rs-152511/v1 |
| 21 | Tarteel Musa et al., 2022 [21] | *Prevalence of depression among medical students in River Nile State Universities 2021* | <https://doi.org/10.12688/f1000research.123648.1> | Included |  | unpublished | https://f1000research.com/articles/11-1168 |
| 22 | Khalid A. Khalil et al. [22] | *Depressive Symptoms Amongst Undergraduate Students in Libya.* | <http://doi.org/10.5281/zenodo.4723954> | Included |  | Published |  |
| 23 | H Essangri et al., 2021[23] | *Predictive factors for impaired mental health among medical students during the early stage of the COVID-19 pandemic in Morocco.* | <https://doi.org/10.4269/ajtmh.20-1302> | Included |  | Published |  |
| 24 | Rammouz et al., 2023 [24] | *Religiosity, stress, and depressive symptoms among nursing and medical students during the middle stage of the COVID-19 pandemic: A cross-sectional study in Morocco.* | <https://doi.org/10.3389/fpsyt.2023.1123356> | Included |  | Published |  |
| 25 | Terasaki, D.J., et al.[25] | *Anger expression, violent behavior, and symptoms of depression among male college students in Ethiopia.* | <https://doi.org/10.1186/1471-2458-9-13> | Excluded | Target population difference | Published |  |
| 26 | Shereen Esmat et al., 2021 [26] | *Prevalence and predictors for depression among medical students during coronavirus disease-19 pandemic: A cross-sectional study.* | <https://doi.org/10.3889/oamjms.2021.7390> | Included |  | Published |  |
| 27 | Ibrahim, A.K., S.J. Kelly, and C. Glazebrook[27] | *Analysis of an Egyptian study on the socioeconomic distribution of depressive symptoms among undergraduates.* | <https://doi.org/10.1007/s00127-011-0400-x> | Excluded | Outcome of interest and Target population difference | Published |  |
| 28 | Mwita M et al., 2020 [28] | *Prevalence of Depression and Suicidality among Medical Students in Mwanza Tanzania; A Cross-Sectional Study.* | <https://dx.doi.org/10.4103/ejpsy.ejpsy_1_20> | Included |  | Published |  |
| 29 | Sserunkuuma, J., et al., 2023 [29] | *Problematic use of the internet, smartphones, and social media among medical students and relationship with depression: An exploratory study.* | <https://doi.org/10.1371/journal.pone.0286424> | Included |  | Published |  |
| 30 | S. H. Mustafa et al., 2022 [30] | *Mental distress among medical students in Khartoum, Sudan 2022.* | <https://doi.org/10.4236/ojpsych.2022.124026> | Included |  | Published |  |
| 31 | Hetolang, L.T. and K. Amone-P’Olak[31] | *The associations between stressful life events and depression among students in a university in Botswana.* | <https://doi.org/10.1177/0081246317711793> | Excluded | Target Population difference | Published |  |
| 32 | Mohamed, E.A.A., et al., 2018 [32] | *Prevalence of depression among medical students in Sudan International University in May 2017–August 2017.* | <http://www.ncbi.nlm.nih/gov/pubmed/27892436> | Included |  | Published |  |
| 33 | Dafaalla, M., et al., 2016 [33] | *Depression, anxiety, and stress in Sudanese medical students: a cross sectional study on role of quality of life and social support.* | DOI:10.12691/education-4-13-4 | Included |  | Published |  |
| 34 | Nubi et al. ,2022 [34] | *Depression, Anxiety, and Stress among Sudanese Medical Students during the COVID-19 Lockdown Period.* | <https://orcid.org/0000-0001-9276-024X> | Included |  | Published |  |
| 35 | Peltzer, K., et al[35]. | *Depression and associated factors among university students in Western Nigeria* | <https://doi.org/10.1007/s00127-006-0068-9> | Excluded | The outcome of interest was not clear  Target Population difference | Published |  |
| 36 | Othieno, C.J., et al.[36] | *Depression among university students in Kenya: Prevalence and sociodemographic correlates.* | <https://doi.org/10.1016/j.jad.2014.04.070> | Excluded | Target Population difference | Published |  |
| 37 | Muriungi, S.K. and D.M. Ndetei[37] | *Effectiveness of psycho-education on depression, hopelessness, suicidality, anxiety and substance use among basic diploma students at Kenya Medical Training College.* | DOI:10.7196/SAJP.401 | Excluded | Target Population difference | Published |  |
| 38 | Makhubela, M.[38] | *Suicide and depression in university students: a possible epidemic*. | <https://doi.org/10.1177/0081246321992179> | Excluded | The outcome of interest was not clear  Target Population difference | published |  |
| 39 | Croock, J., et al.[39] | *Probable depression and its correlates among undergraduate students in Johannesburg, South Africa.* | <https://doi.org/10.3389/fpsyt.2023.1018197> | Excluded | Outcome measurement was not clear  Target Population difference | Published |  |
| 40 | Bantjes, J.R., et al.[40] | *Symptoms of posttraumatic stress, depression, and anxiety as predictors of suicidal ideation among South African university students.* | <https://doi.org/10.1080/07448481.2016.1178120> | Excluded | Target Population difference | Published |  |
| 41 | Oppong Asante, K. and J. Andoh-Arthur[41] | *Prevalence and determinants of depressive symptoms among university students in Ghana.* | <https://doi.org/10.1016/j.jad.2014.09.025> | Excluded | Target Population difference | Published |  |
| 42 | Shawahna, R., et al.[42] | *Prevalence and factors associated with depressive and anxiety symptoms among Palestinian medical students.* | <https://doi.org/10.1186/s12888-020-02658-1> | Excluded | Target Population difference  Not African study | Published |  |
| 43 | Santander-Hernández, F.M., et al.[43] | *Smartphone overuse, depression & anxiety in medical students during the COVID-19 pandemic.* | <https://doi.org/10.1371/journal.pone.0273575> | Excluded | Not African study | Published |  |
| 44 | Lugata, S., et al.[44] | *Symptoms and predictors of depression among university students in the Kilimanjaro region of Tanzania: a cross-sectional study.* | <https://doi.org/10.1080/09638237.2020.1793129> | Excluded | Target Population difference | Published |  |
| 45 | Moutinho, I.L.D., et al.[45] | *Depression, stress and anxiety in medical students: A cross-sectional comparison between students from different semesters.* | <https://doi.org/10.1590/1806-9282.63.01.21> | Excluded | The outcome of interest not much  Not African study | Published |  |
| 46 | Romo-Nava, F., et al.[46] | *Major depressive disorder in Mexican medical students and associated factors: A focus on current and past abuse experiences.* | <https://doi.org/10.1016/j.jad.2018.11.083> | Excluded | The outcome of interest not much | Published |  |
| 47 | López, R.B., et al. [47] | *Relationship between personality organization and the prevalence of symptoms of depression, anxiety and stress among university students in health careers in the Region of Coquimbo, Chile.* | <https://doi.org/10.1016/j.rcpeng.2017.09.003> | Excluded | Target Population difference | Published |  |
| 48 | Chan, H.W.Q. and C.F.R. Sun.[48] | *Irrational beliefs, depression, anxiety, and stress among university students in Hong Kong.* | <https://doi.org/10.1080/07448481.2019.1710516> | Excluded | The tool not clear | Published |  |
| 49 | Swed, S., et al.[49] | *Stigmatizing attitudes towards depression among university students in Syria.* | <https://doi.org/10.1371/journal.pone.0273483> | Excluded | Target Population difference  Not African study | Published |  |
| 50 | Piscoya-Tenorio, et al. [50] | *Prevalence and Factors Associated with Anxiety and Depression in Peruvian Medical Students* | <https://doi.org/10.3390/ijerph20042907> | Excluded | Not African study | Published |  |
| 51 | Tadi, N.F., et al.[51] | *Sex differences in depression and anxiety symptoms: measurement invariance, prevalence, and symptom heterogeneity among university students in South Africa* | <https://doi.org/10.3389/fpsyg.2022.873292> | Excluded | Target Population difference  Outcome of interest not clearly reported |  |  |
| 52 | Hernández-Yépez, P.J., et al.[52] | *Factors associated with anxiety, depression, and stress in Peruvian university students during the COVID-19 pandemic.* | <https://doi.org/10.3390/ijerph192114591> | Excluded | Target Population difference | Published |  |
| 53 | Džubur, A., et al.[53] | *Depressive Symptoms Among Sarajevo University Students: Prevalence and Socio-Demographic Correlations.* | <https://doi.org/10.5644/ama2006-124.227> | Excluded | Target Population difference | Published |  |
| 54 | Wang, Q., et al.[54] | *Anxiety and depression and their interdependent influencing factors among medical students in Inner Mongolia: the cross-sectional survey.* | <https://doi.org/10.1186/s12909-022-03839-0> | Excluded | Not African study | Published |  |
| 55 | Mall, S., et al.[55] | *The relationship between childhood adversity, recent stressors, and depression in college students attending a South African university.* | <https://doi.org/10.1186/s12888-017-1583-9> | Excluded | Target Population difference | Published |  |
| 56 | Malebana, C.M., et al.[56] | *Depression, Anxiety and Substance Use Among Undergraduate Students at North-West University, South Africa.* | <https://doi.org/10.5539/gjhs.v11n13p85> | Excluded | Target Population difference | Published |  |
| 57 | Zhong, Y., et al.[57] | *Social Support, Health Literacy and Depressive Symptoms among Medical Students* | <https://doi.org/10.3390/ijerph18020633> | Excluded | Not African study | Published |  |
| 58 | Desouky, D.E.et al.[58] | *Migraine, tension-type headache, and depression among Saudi female students in Taif University* | <https://doi.org/10.1186/s42506-019-0008-7> | Excluded | Target Population difference | Published |  |
| 59 | Baskaya, O.,et al.[59] | *The relationship between social achievement goals and self-esteem, depression and anxiety among medical school students.* | <https://doi.org/10.4103/njcp.njcp_69_23> | Excluded | Not African study | Published |  |
| 60 | Juanico-Morales, L. and E. Nava-Aguilera[60] | *Depression and associated factors in medical students in Acapulco during the COVID-19 pandemic: A cross-sectional study.* | <https://doi.org/10.1371/journal.pone.0285903> | Excluded | Not African study | Published |  |
| 61 | Othieno, C.J., et al.[61] | *Risky HIV sexual behaviour and depression among University of Nairobi students* | <https://doi.org/10.1186/s12991-015-0054-2> | Excluded | Target Population difference | Published |  |
| 62 | Dapaah, J.M. and H.A.F. Amoako.[62] | *The causes of depression among university students and its effects on their academic life in the Kwame Nkrumah University of Science and Technology, Kumasi, Ghana.* | <https://doi.org/10.31686/ijier.Vol7.Iss6.1504> | Excluded | Target Population difference | Published |  |
| 63 | Tashiro, T., et al.[63] | *Association between sedentary behavior and depression among Japanese medical students during the COVID-19 pandemic: a cross-sectional online survey* | <https://doi.org/10.1186/s12888-022-03997-x> | Excluded | Not African study  The outcome of interest not clearly reported | Published |  |
| 64 | Pillay, A.L., J.D., et al.[64] | *Depressive symptoms in first year students at a rural South African University.* | <https://doi.org/10.1016/j.jad.2019.11.094> | Excluded | Target Population difference | Published |  |
| 65 | Isara, A.R., O.I. Nwokoye, and A.O. Odaman.[65] | *Prevalence and risk factors of depression among undergraduate medical students in a Nigerian university.* | <https://doi.org/10.4314/gmj.v56i4.9> | Excluded | Target Population difference | Published |  |
| 66 | Lu, L., et al.[66] | *Association of Covid-19 pandemic-related stress and depressive symptoms among international medical students.* | <https://doi.org/10.1186/s12888-021-03671-8> | Excluded | Not African study | Published |  |
| 67 | Najjuka, S.M., et al.[67] | *Depression, anxiety, and stress among Ugandan university students during the COVID-19 lockdown: an online survey.* | <https://doi.org/10.4314/ahs.v21i4.6> | Excluded | Target Population difference | Published |  |
| 68 | Ahmed, G., et al.[68] | *Prevalence and associated factors of depression among Jimma University students. A cross-sectional study.* | <https://doi.org/10.1186/s13033-020-00384-5> | Excluded | Target Population difference | Published |  |
| 69 | Ocal, N.U., M. Kilic, and G. Uslukilic.[69] | *The relationship between university students' depression, anxiety, and stress with positivity attitudes and the COVID-19 pandemic.* | <https://doi.org/10.4103/njcp.njcp_135_22> | Excluded | Target Population difference  Not African study | Published |  |
| 70 | Chumakov, E., et al.[70] | *The impact of covid-19: Anxiety, depression, and wellbeing among medical students.* | <https://doi.org/10.1177/00207640221121717> | Excluded | Not African study | Published |  |
| 71 | Sakai, M., et al.[71] | *Depression and anxiety among nursing students during the COVID-19 pandemic in Tohoku region, Japan: A cross-sectional survey.* | <https://doi.org/10.1111/jjns.12483> | Excluded | Target Population difference | Published |  |
| 72 | Aveiro-Róbalo, T.R.,et al.[72] | *Prevalence and Associated Factors of Depression, Anxiety, and Stress in University Students in Paraguay during the COVID-19 Pandemic.* | <https://doi.org/10.3390/ijerph191912930> | Excluded | Target Population difference | Published |  |
| 73 | Rudenstine, S., et al.[73] | *Depression and Anxiety During the COVID-19 Pandemic in an Urban, Low-Income Public University Sample.* | <https://doi.org/10.1002/jts.22600> | Excluded | Target Population difference | Published |  |
| 74 | Guo, Y., et al.[74] | *Depression and anxiety of medical students at Kunming Medical University during COVID-19: A cross-sectional survey.* | <https://doi.org/10.3389/fpubh.2022.957597> | Excluded | Not African study | Published |  |
| 75 | Shao, R., et al.[75] | *Prevalence of depression and anxiety and correlations between depression, anxiety, family functioning, social support and coping styles among Chinese medical students.* | <https://doi.org/10.1186/s40359-020-00402-8> | Excluded | Not African study | Published |  |
| 76 | Al-Khani, A.M., et al.[76] | *A cross-sectional survey on sleep quality, mental health, and academic performance among medical students in Saudi Arabia.* | <https://doi.org/10.1186/s13104-019-4713-2> | Excluded | Not African study | Published |  |
| 77 | Nikolic, A., et al.[77] | *Smartphone addictions sleep quality, depression, anxiety, and stress among medical students.* | <https://doi.org/10.3389/fpubh.2023.1252371> | Excluded | Not African study | Published |  |
| 78 | Thapa, B., et al.[78] | *Predictors of Depression and Anxiety among Medical Students.* | <https://doi.org/10.33314/jnhrc.v21i1.4514> | Excluded | Not African study | Published |  |
| 79 | Shofler, D.W., et al.[79] | *Prevalence of Depression in Podiatric Medical Students.* | <https://doi.org/10.7547/18-108> | Excluded | Not African study | Published |  |
| 80 | Latifeh, Y., et al.[80] | *Prevalence of internet addiction among Syrian undergraduate medical students.* | <https://doi.org/10.1097/md.0000000000032261> | Excluded | Not African study | Published |  |
| 81 | Iqbal, S., et al.[81] | *Stress, anxiety and depression among medical undergraduate students and their socio-demographic correlates.* | <https://doi.org/10.4103/0971-5916.156571> | Excluded | Not African study | Published |  |
| 82 | Tareq, S.R., et al.[82] | *Depression among Medical Students of Bangladesh.* | Not Found | Excluded | Not African study | Published |  |
| 83 | Shrestha, N., et al.[83] | *Prevalence of Depression among Medical Students of a Tertiary Care Teaching Hospital* | <https://doi.org/10.31729/jnma.4738> | Excluded |  | Published |  |
| 84 | Gan, G.G. and H. Yuen Ling.[84] | *Anxiety, depression and quality of life of medical students in Malaysia.* | Not Found | Excluded | Not African study | Published |  |
| 85 | Azad, N., et al.[85] | *Anxiety And Depression In Medical Students Of A Private Medical College.* | Not Found | Excluded | Study Area difference | Published |  |
| 86 | Khatri, B., et al.[86] | *Depression among Medical Students of a Medical College: A Descriptive Cross-sectional Study.* | <https://doi.org/10.31729/jnma.7869> | Excluded | Not African study | Published |  |
| 87 | Shamsuddin, K., et al.[87] | *Correlates of depression, anxiety and stress among Malaysian university students.* | <https://doi.org/10.1016/j.ajp.2013.01.014> | Excluded | Variation in target population | Published |  |
| 88 | Capdevila-Gaudens, P., et al.[88] | *Depression, anxiety, burnout and empathy among Spanish medical students.* | <https://doi.org/10.1371/journal.pone.0260359> | Excluded | Not African study | published |  |
