# Supplementary material for: Prevalence of depression among medical students in Africa: Systematic review and meta-analysis
Source: PLoS One. 2024 Dec 26;19(12):e0312281. doi: 10.1371/journal.pone.0312281 (PMC11670985; doi:10.1371/journal.pone.0312281)
Supplement: S2 Table — (DOCX) [file pone.0312281.s005.docx]

**S2 Table:** A table of all data extracted from the primary research sources for the systematic review and/or meta-analysis

| S/N | Publication year | Author/s | Tool used | Country | Study design | Sample | prevalence | Response rate | Name of data extractor | Data of data extraction | Eligibility confirmation |
| --- | --- | --- | --- | --- | --- | --- | --- | --- | --- | --- | --- |
| 1 | 2019 | Kebede et al. | HADS | Ethiopia | Cross-sectional | 273 | 51.30 | 98.50 | HKA and AWA | April, 2023-June 2023 | Eligible |
| 2 | 2020 | Dagnew et al. | BDI | Ethiopia | Cross-sectional | 383 | 34.73 | 97.70 | HKA and AWA | April, 2023-June 2023 | Eligible |
| 3 | 2020 | S van der Walt et al. | HADS | South Africa | Cross-sectional | 473 | 25.00 | 100.00 | HKA and AWA | April, 2023-June 2023 | Eligible |
| 4 | 2017 | Bawo O. James et al. | HADS | Nigeria | Cross-sectional | 623 | 21.30 | 98.10 | HKA and AWA | April, 2023-June 2023 | Eligible |
| 5 | 2020 | Joshua Falade et al. | HADS | Nigeria | Cross-sectional | 944 | 14.30 | 97.80 | HKA and AWA | April, 2023-June 2023 | Eligible |
| 6 | 2020 | M. Barrimi et al. | BDI | Morocco | Cross-sectional | 605 | 10.40 | 100.00 | HKA and AWA | April, 2023-June 2023 | Eligible |
| 7 | 2020 | Mboya et al. | SRQ-20 | Tanzania | Cross-sectional | 203 | 14.30 | 100.00 | HKA and AWA | April, 2023-June 2023 | Eligible |
| 8 | 2020 | Olum et al | PHQ-9 | Uganda | Cross-sectional | 331 | 21.50 | 94.00 | HKA and AWA | April, 2023-June 2023 | Eligible |
| 9 | 2017 | Ngasa et al | PHQ-9 | Cameroon | Cross-sectional | 618 | 30.60 | 90.00 | HKA and AWA | April, 2023-June 2023 | Eligible |
| 10 | 2018 | Njim T, et al | PHQ-9 | Cameroon | Cross-sectional | 413 | 66.34 | 82.60 | HKA, CKM AWA | April, 2023-June 2023 | Eligible |
| 11 | 2021 | Edmund Ndudi Ossai et al | BDI | Nigeria | Cross-sectional | 522 | 26.60 | 100.00 | HKA and AWA | April, 2023-June 2023 | Eligible |
| 12 | 2019 | El-Gilany et al | BDI | Egypt | Cross-sectional | 900 | 25.20 | 100.00 | HKA and AWA | April, 2023-June 2023 | Eligible |
| 13 | 2021 | C. E. NWACHUKWU ET AL. | HADS | Nigeria | Cross-sectional | 690 | 10.10 | 100.00 | HKA and AWA | April, 2023-June 2023 | Eligible |
| 14 | 2017 | Mohamed Fawzy | DASS-21 | Egypt | Cross-sectional | 700 | 65.00 | 100.00 | HKA and AWA | April, 2023-June 2023 | Eligible |
| 15 | 2016 | Narushni Pillay et al. | DASS-21 | South Africa | Cross-sectional | 230 | 15.60 | NA | HKA and AWA | April, 2023-June 2023 | Eligible |
| 16 | 2021 | Uzoechi Eze Chikezie et al. | DASS-21 | Nigeria | Cross-sectional | 243 | 25.5 | 100.00 | HKA and AWA | April, 2023-June 2023 | Eligible |
| 17 | 2020 | Wafaa et al. | DASS-21 | Egypt | Cross-sectional | 390 | 45.1 | 100.00 | HKA and AWA | April, 2023-June 2023 | Eligible |
| 18 | 2021 | Sherif RF et al. | PHQ-9 | Lybia | Cross-sectional | 170 | 45 | 100.00 | HKA and AWA | April, 2023-June 2023 | Eligible |
| 19 | 2021 | Suraj, et al. | SRQ-20 | Nigeria | Cross-sectional | 279 | 15.1 | 100.00 | HKA and AWA | April, 2023-June 2023 | Eligible |
| 20 | 2021 | Leta Melaku et al. | DASS-21 | Ethiopia | Cross-sectional | 260 | 53 | 98.10 | HKA and AWA | April, 2023-June 2023 | Eligible |
| 21 | 2022 | Tarteel Musa et al. | HADS | Sudan | Cross-sectional | 355 | 78 | 100.00 | HKA and AWA | April, 2023-June 2023 | Eligible |
| 22 | 2019 | Khalid A. Khalil | PHQ-9 | Lybia | Cross-sectional | 1300 | 45 | 74.60 | HKA and AWA | April, 2023-June 2023 | Eligible |
| 23 | 2021 | H Essangri et al. | BDI | Morocco | Cross-sectional | 549 | 74.7 | 100.00 | HKA and AWA | April, 2023-June 2023 | Eligible |
| 24 | 2023 | Rammouz et al. | BDI | Morocco | Cross-sectional | 92 | 41.3 | 91.40 | HKA and AWA | April, 2023-June 2023 | Eligible |
| 25 | 2021 | Shereen Esmat et al. | BDI | Egypt | Cross-sectional | 238 | 38.2 | 79.30 | HKA and AWA | April, 2023-June 2023 | Eligible |
| 26 | 2020 | Mwita M et al. | PHQ-9 | Tanzania | Cross-sectional | 353 | 41.36 | 100.00 | HKA and AWA | April, 2023-June 2023 | Eligible |
| 27 | 2023 | Sserunkuuma, J., et al | PHQ-9 | Uganda | Cross-sectional | 269 | 16.73 | 100.00 | HKA and AWA | April, 2023-June 2023 | Eligible |
| 28 | 2020 | S. H. Mustafa et al. | SRQ-20 | Sudan | Cross-sectional | 432 | 55.8 | 100.00 | HKA and AWA | April, 2023-June 2023 | Eligible |
| 29 | 2018 | Mohamed, E.A.A.,et al. | PHQ-9 | Sudan | Cross-sectional | 440 | 67 | 100.00 | HKA and AWA | April, 2023-June 2023 | Eligible |
| 30 | 2016 | Dafaalla, M., et al. | DASS-21 | Sudan | Cross-sectional | 487 | 53.4 | 97.40 | HKA and AWA | April, 2023-June 2023 | Eligible |
| 31 | 2022 | Nubi et al. | DASS-21 | Sudan | Cross-sectional | 1058 | 75 | 99.90 | HKA and AWA | April, 2023-June 2023 | Eligible |

**Factors associated with depression**

| Being Female medical student as a factor for depression | | | | | | | | | | | | | | | | | | | | | | | | | | | | | | | | | | | | | | | | | | | | | | | | | | | | | | | | | | | | | | | |
| --- | --- | --- | --- | --- | --- | --- | --- | --- | --- | --- | --- | --- | --- | --- | --- | --- | --- | --- | --- | --- | --- | --- | --- | --- | --- | --- | --- | --- | --- | --- | --- | --- | --- | --- | --- | --- | --- | --- | --- | --- | --- | --- | --- | --- | --- | --- | --- | --- | --- | --- | --- | --- | --- | --- | --- | --- | --- | --- | --- | --- | --- | --- | --- |
| S/N | publication year | | | | Author/s | | | | | | Tool used | | | | | Country | | | | | study design | | | | | | | sample size | | | | | | AOR | | | | UBCI | | | | | | | LBCI | | | | | logor | | | | | | selogor | | | Name of Data extractor | | | Date of data extraction | Eligibility confirmation |
| 1 | 2019 | | | | Kebede et al. | | | | | | HADS | | | | | Ethiopia | | | | | Cross-sectional | | | | | | | 273 | | | | | | 0.99 | | | | 1.75 | | | | | | | 0.57 | | | | | -0.01 | | | | | | 1.60 | | | AFZ and ZWB | | | April, 2023-June 2023 | eligible |
| 2 | 2020 | | | | S van der Walt et al. | | | | | | HADS | | | | | South Africa | | | | | Cross-sectional | | | | | | | 473 | | | | | | 3.7 | | | | 6.8 | | | | | | | 2 | | | | | 1.31 | | | | | | 6.29 | | | AFZ and ZWB | | | April, 2023-June 2023 | eligible |
| 3 | 2020 | | | | M. Barrimi et al. | | | | | |  | | | | | Morocco | | | | | Cross-sectional | | | | | | | 605 | | | | | | 0.16 | | | | 0.33 | | | | | | | 0.07 | | | | | -1.83 | | | | | | 0.31 | | | AFZ and ZWB | | | April, 2023-June 2023 | eligible |
| 4 | 2020 | | | | Olum et al | | | | | | PHQ9 | | | | | Uganda | | | | | Cross-sectional | | | | | | | 331 | | | | | | 1.11 | | | | 2 | | | | | | | 0.59 | | | | | 0.10 | | | | | | 1.85 | | | AFZ and ZWB | | | April, 2023-June 2023 | eligible |
| 5 | 2017 | | | | Ngasa et al | | | | | | PHQ-9 | | | | | Cameroon | | | | | Cross-sectional | | | | | | | 618 | | | | | | 1.59 | | | | 2.37 | | | | | | | 1.06 | | | | | 0.46 | | | | | | 2.10 | | | AFZ and ZWB | | | April, 2023-June 2023 | eligible |
| 6 | 2019 | | | | El-Gilany et al | | | | | | BDI | | | | | Egypt | | | | | Cross-sectional | | | | | | | 900 | | | | | | 0.59 | | | | 0.91 | | | | | | | 0.38 | | | | | -0.53 | | | | | | 0.81 | | | AFZ and ZWB | | | April, 2023-June 2023 | eligible |
| 7 | 2021 | | | | C. E. NWACHUKWU ET AL. | | | | | |  | | | | | Nigeria | | | | | Cross-sectional | | | | | | | 690 | | | | | | 1.2 | | | | 1.89 | | | | | | | 0.79 | | | | | 0.18 | | | | | | 1.69 | | | AFZ and ZWB | | | April, 2023-June 2023 | eligible |
| 8 | 2020 | | | | Wafaa et al. | | | | | | DASS 21 | | | | | Egypt | | | | | Cross-sectional | | | | | | | 390 | | | | | | 1.7 | | | | 2.71 | | | | | | | 1.04 | | | | | 0.53 | | | | | | 2.44 | | | AFZ and ZWB | | | April, 2023-June 2023 | eligible |
| 9 | 2021 | | | | Suraj, et al. | | | | | |  | | | | | Nigeria | | | | | Cross-sectional | | | | | | | 279 | | | | | | 2.88 | | | | 6.33 | | | | | | | 1.21 | | | | | 1.06 | | | | | | 6.02 | | | AFZ and ZWB | | | April, 2023-June 2023 | eligible |
| 10 | 2021 | | | | Leta Melaku et al. | | | | | | Depression, Anxiety, Stress Scale(DASS-21) | | | | | Ethiopia | | | | | Cross-sectional | | | | | | | 260 | | | | | | 1.33 | | | | 2.94 | | | | | | | 1.05 | | | | | 0.29 | | | | | | 2.67 | | | AFZ and ZWB | | | April, 2023-June 2023 | eligible |
| 11 | 2021 | | | | Shereen Esmat et al. | | | | | | Beck’s Depression Inventory | | | | | Egypt | | | | | Cross-sectional | | | | | | | 238 | | | | | | 2.44 | | | | 5.21 | | | | | | | 1.14 | | | | | 0.89 | | | | | | 4.92 | | | AFZ and ZWB | | | April, 2023-June 2023 | eligible |
| 12 | 2020 | | | | Mwita M et al. | | | | | | (PHQ-9) | | | | | Tanzania | | | | | Cross-sectional | | | | | | | 353 | | | | | | 1.43 | | | | 2 | | | | | | | 0.83 | | | | | 0.36 | | | | | | 1.79 | | | AFZ and ZWB | | | April, 2023-June 2023 | eligible |
| Year of study (first, second, third, fourth, fifth and sixth) | | | | | | | | | | | | | | | | | | | | | | | | | | | | | | | | | | | | | | | | | | | | | | | | | | | | | | | | | | | | | | |  |
| 1 | 2019 | Kebede et al. | | | | | | | HADS | | | | | | Ethiopia | | | | | Cross-sectional | | | | | | 273 | | | | | 1.63 | | | | 6.26 | | | | | | 1.43 | | | | | | 0.49 | | | | | | | | | 5.89 | | HKA, CKM and AWA | | | April, 2023-June 2023 | | eligible |
| 2 | 2020 | Olum et al | | | | | | | PHQ-9 | | | | | | Uganda | | | | | Cross-sectional | | | | | | 331 | | | | | 1.43 | | | | 5 | | | | | | 0.5 | | | | | | 0.36 | | | | | | | | | 4.87 | | HKA, CKM and AWA | | | April, 2023-June 2023 | | eligible |
| 3 | 2021 | Leta Melaku et al. | | | | | | | DASS-21 | | | | | | Ethiopia | | | | | Cross-sectional | | | | | | 260 | | | | | 1.91 | | | | 4.28 | | | | | | 0.85 | | | | | | 0.65 | | | | | | | | | 4.06 | | HKA, CKM and AWA | | | April, 2023-June 2023 | | eligible |
| 4 | 2020 | Mwita M et al. | | | | | | | PHQ-9 | | | | | | Tanzania | | | | | Cross-sectional | | | | | | 353 | | | | | 1.43 | | | | 5 | | | | | | 0.53 | | | | | | 0.36 | | | | | | | | | 4.86 | | HKA, CKM and AWA | | | April, 2023-June 2023 | | eligible |
| 5 | 2021 | Suraj, et al. | | | | | | | SRQ-20 | | | | | | Nigeria | | | | | Cross-sectional | | | | | | 279 | | | | | 2.88 | | | | 4.67 | | | | | | 1.72 | | | | | | 1.06 | | | | | | | | | 4.23 | | HKA, CKM and AWA | | | April, 2023-June 2023 | | eligible |
| Second year | | | | | | | | | | | | | | | | | | | | | | | | | | | | | | | | | | | | | | | | | | | | | | | | | | | | | | | | | | | | | | |  |
| 1 | 2019 | Kebede et al. | | | | | | | HADS | | | | | Ethiopia | | | | | Cross-sectional | | | | | | | 273 | | | | | 1.39 | | | | 5.18 | | | | | | 1.57 | | | | | | 0.33 | | | | | | 4.78 | | | | | HKA, CKM and AWA | | | | April, 2023-June 2023 | eligible |
| 2 | 2020 | Olum et al | | | | | | | PHQ-9 | | | | | Uganda | | | | | Cross-sectional | | | | | | | 331 | | | | | 1.6 | | | | 3.8 | | | | | | 0.7 | | | | | | 0.47 | | | | | | 3.62 | | | | | HKA, CKM and AWA | | | | April, 2023-June 2023 | eligible |
| 3 | 2021 | Leta Melaku et al. | | | | | | | DASS-21 | | | | | Ethiopia | | | | | Cross-sectional | | | | | | | 260 | | | | | 4.5 | | | | 10.73 | | | | | | 1.88 | | | | | | 1.50 | | | | | | 10.25 | | | | | HKA, CKM and AWA | | | | April, 2023-June 2023 | eligible |
| 4 | 2020 | Mwita M et al. | | | | | | | PHQ-9 | | | | | Tanzania | | | | | Cross-sectional | | | | | | | 353 | | | | | 0.24 | | | | 0.53 | | | | | | 0.11 | | | | | | -1.42 | | | | | | 0.50 | | | | | HKA, CKM and AWA | | | | April, 2023-June 2023 | eligible |
| 5 | 2021 | Suraj, et al. | | | | | | | SRQ-20 | | | | | Nigeria | | | | | Cross-sectional | | | | | | | 279 | | | | | 5.11 | | | | 9.46 | | | | | | 3.23 | | | | | | 1.63 | | | | | | 8.64 | | | | | HKA, CKM and AWA | | | | April, 2023-June 2023 | eligible |
| Third year | | | | | | | | | | | | | | | | | | | | | | | | | | | | | | | | | | | | | | | | | | | | | | | | | | | | | | | | | | | | | | |  |
| 1 | 2019 | | | Kebede et al. | | | | | HADS | | | | | Ethiopia | | | | | Cross-sectional | | | | | | 273 | | | | | 0.5 | | | 1.65 | | | | | | | 0.16 | | | | -0.69 | | | | | | | 1.61 | | | | HKA, CKM and AWA | | | | | April, 2023-June 2023 | | | eligible |
| 2 | 2020 | | | Olum et al | | | | | PHQ-9 | | | | | Uganda | | | | | Cross-sectional | | | | | | 331 | | | | | 1.3 | | | 3.6 | | | | | | | 0.5 | | | | 0.26 | | | | | | | 3.47 | | | | HKA, CKM and AWA | | | | | April, 2023-June 2023 | | | eligible |
| 3 | 2021 | | | Leta Melaku et al. | | | | | DASS-21 | | | | | Ethiopia | | | | | Cross-sectional | | | | | | 260 | | | | | 4.85 | | | 12.19 | | | | | | | 1.93 | | | | 1.58 | | | | | | | 11.70 | | | | HKA, CKM and AWA | | | | | April, 2023-June 2023 | | | eligible |
| 4 | 2020 | | | Mwita M et al. | | | | | PHQ-9 | | | | | Tanzania | | | | | Cross-sectional | | | | | | 353 | | | | | 0.36 | | | 0.77 | | | | | | | 0.16 | | | | -1.02 | | | | | | | 0.73 | | | | HKA, CKM and AWA | | | | | April, 2023-June 2023 | | | eligible |
| 5 | 2021 | | | Suraj, et al. | | | | | SRQ-20 | | | | | Nigeria | | | | | Cross-sectional | | | | | | 279 | | | | | 9.37 | | | 14.78 | | | | | | | 3.89 | | | | 2.24 | | | | | | | 13.78 | | | |  | | | | | April, 2023-June 2023 | | | eligible |
| Fourth Year | | | | | | | | | | | | | | | | | | | | | | | | | | | | | | | | | | | | | | | | | | | | | | | | | | | | | | | | | | | | | | |  |
| 1 | 2019 | | | Kebede et al. | | | | HADS | | | | | | Ethiopia | | | | | Cross-sectional | | | | | 273 | | | | | | 0.52 | | | 1.68 | | | | 0.16 | | | | | | | -0.65 | | | | | | | 1.49 | | | | HKA, CKM and AWA | | | | | | | April, 2023-June 2023 | eligible |
| 2 | 2020 | | | Olum et al | | | | PHQ-9 | | | | | | Uganda | | | | | Cross-sectional | | | | | 331 | | | | | | 0.4 | | | 1.2 | | | | 0.1 | | | | | | | -0.92 | | | | | | | 1.10 | | | | HKA, CKM and AWA | | | | | | | April, 2023-June 2023 | eligible |
| 3 | 2021 | | | Leta Melaku et al. | | | | DASS-21 | | | | | | Ethiopia | | | | | Cross-sectional | | | | | 260 | | | | | | 2.34 | | | 5.22 | | | | 1.05 | | | | | | | 0.85 | | | | | | | 4.09 | | | | HKA, CKM and AWA | | | | | | | April, 2023-June 2023 | eligible |
| 4 | 2020 | | | Mwita M et al. | | | | PHQ-9 | | | | | | Tanzania | | | | | Cross-sectional | | | | | 353 | | | | | | 0.56 | | | 1.43 | | | | 0.23 | | | | | | | -0.58 | | | | | | | 1.18 | | | | HKA, CKM and AWA | | | | | | | April, 2023-June 2023 | eligible |
| 5 | 2021 | | | Suraj, et al. | | | | SRQ-20 | | | | | | Nigeria | | | | | Cross-sectional | | | | | 279 | | | | | | 8.52 | | | 16.32 | | | | 2.96 | | | | | | | 2.14 | | | | | | | 13.09 | | | | HKA, CKM and AWA | | | | | | | April, 2023-June 2023 | eligible |
| Fifth year | | | | | | | | | | | | | | | | | | | | | | | | | | | | | | | | | | | | | | | | | | | | | | | | | | | | | | | | | | | | | | |  |
| 1 | 2019 | | | Kebede et al. | | HADS | | | | | | | | Ethiopia | | | | | Cross-sectional | | | | | 273 | | | | | | 1.9 | | | 8.81 | | | | 0.41 | | | | | | 0.64 | | | | | | 2.15 | | | | | HKA, CKM and AWA | | | | | | | | April, 2023-June 2023 | eligible |
| 2 | 2020 | | | Olum et al | | PHQ-9 | | | | | | | | Uganda | | | | | Cross-sectional | | | | | 331 | | | | | | 0.7 | | | 2 | | | | 0.2 | | | | | | -0.36 | | | | | | 0.50 | | | | | HKA, CKM and AWA | | | | | | | | April, 2023-June 2023 | eligible |
| 3 | 2021 | | | Leta Melaku et al. | | DASS-21 | | | | | | | | Ethiopia | | | | | Cross-sectional | | | | | 260 | | | | | | 2.01 | | | 4.94 | | | | 0.82 | | | | | | 0.7 | | | | | | 1.10 | | | | | HKA, CKM and AWA | | | | | | | | April, 2023-June 2023 | eligible |
| 4 | 2020 | | | Mwita M et al. | | PHQ-9 | | | | | | | | Tanzania | | | | | Cross-sectional | | | | | 353 | | | | | | 0.7 | | | 1.9 | | | | 0.2 | | | | | | -0.36 | | | | | | 0.43 | | | | | HKA, CKM and AWA | | | | | | | | April, 2023-June 2023 | eligible |
| 5 | 2021 | | | Suraj, et al. | | SRQ-20 | | | | | | | | Nigeria | | | | | Cross-sectional | | | | | 279 | | | | | | 2.87 | | | 0.13 | | | |  | | | | | | -0.41 | | | | | | 0.70 | | | | | HKA, CKM and AWA | | | | | | | | April, 2023-June 2023 | eligible |
| Clinical stage of students (preclinical and clinical) | | | | | | | | | | | | | | | | | | | | | | | | | | | | | | | | | | | | | | | | | | | | | | | | | | | | | | | | | | | | | | |  |
| 1 | 2020 | | S van der Walt et al. | | | | | | | HADS | | South Africa | | | | | | Cross-sectional | | | | | 473 | | | | | | 1.2 | | | 2 | | | | 0.7 | | | | | | 0.18 | | | | | | 1.82 | | | | | | AFZ and ZWB | | | | | | | | April, 2023-June 2023 | eligible |
| 2 | 2017 | | Ngasa et al | | | | | | | PHQ-9 | | Cameroon | | | | | | Cross-sectional | | | | | 618 | | | | | | 4.26 | | | 6.71 | | | | 2.71 | | | | | | 1.45 | | | | | | 6.02 | | | | | | AFZ and ZWB | | | | | | | | April, 2023-June 2023 | eligible |
| 3 | 2019 | | El-Gilany et al | | | | | | | BDI | | Egypt | | | | | | Cross-sectional | | | | | 900 | | | | | | 0.67 | | | 0.91 | | | | 0.46 | | | | | | -0.4 | | | | | | 0.79 | | | | | | AFZ and ZWB | | | | | | | | April, 2023-June 2023 | eligible |
| 4 | 2021 | | C. E. NWACHUKWU ET AL. | | | | | | | HADS | | Nigeria | | | | | | Cross-sectional | | | | | 690 | | | | | | 4.1 | | | 7.25 | | | | 2.36 | | | | | | 1.41 | | | | | | 6.65 | | | | | | AFZ and ZWB | | | | | | | | April, 2023-June 2023 | eligible |
| Social support of medical students | | | | | | | | | | | | | | | | | | | | | | | | | | | | | | | | | | | | | | | | | | | | | | | | | | | | | | | | | | | | | | |  |
| 1 | 2019 | | Kebede et al. | | | | HADS | | | | | | Ethiopia | | | | Cross-sectional | | | | | 273 | | | | | 1.32 | | | | | 2.73 | | | | | | | 0.63 | | | | | | | 0.28 | | | | | | 2.50 | | | | | AFZ and ZWB | | | | | April, 2023-June 2023 | eligible |
| 2 | 2021 | | Suraj, et al. | | | | SRQ-20 | | | | | | Nigeria | | | | Cross-sectional | | | | | 279 | | | | | 1.46 | | | | | 2.39 | | | | | | | 0.89 | | | | | | | 0.38 | | | | | | 1.82 | | | | | AFZ and ZWB | | | | | April, 2023-June 2023 | eligible |
| 3 | 2020 | | Mboya et al. | | | | SRQ-20 | | | | | | Tanzania | | | | Cross-sectional | | | | | 402 | | | | | 4.55 | | | | | 9.1 | | | | | | | 2.22 | | | | | | | 1.52 | | | | | | 9.1 | | | | | AFZ and ZWB | | | | | April, 2023-June 2023 | eligible |

Note: AWA; Abere Woretaw Azagew, AFZ; Alebachew Ferede Zegeye, CKM; Chilot Kassa Mekonnen, HKA; Hailemichael Kindie Abate, ZWB; Zerko Wako Beko
