# Supplementary material for: Prevalence of depression among medical students in Africa: Systematic review and meta-analysis
Source: PLoS One. 2024 Dec 26;19(12):e0312281. doi: 10.1371/journal.pone.0312281 (PMC11670985; doi:10.1371/journal.pone.0312281)
Supplement: S3 Table — (DOCX) [file pone.0312281.s006.docx]

|  | **Domain of certainty of included articles** | | | | | | **Factor that increase certainty included articles** | | | **Overall quality** |
| --- | --- | --- | --- | --- | --- | --- | --- | --- | --- | --- |
| Number of studies | Study design | Publication bias | Indirectness | Inconsistency /heterogeneity | Imprecision | Risk bias | Magnitude of effect | Dose response gradient | Effect of confounding variables |  |
| 31 | Observational studies (evidence of certainty assessment started at low because of the design) | Not serious (No evidence of publication bias based on funnel plot and egger’s test) | Not serious (all studies the outcome variable objectively) | Serious (significant heterogeneity detected) | Not serious (all included studies have good sample size, narrow confidence interval of the estimate) | Serious (few number of studies included, the method employed in the study is cross-sectional, prone to bias) | Large magnitude of effect is observed in this meta-analysis | Not applicable for observational studies | All included studies have  controlled the effect of confounding | Low |

**S3 Table**: Grade Score of the included studies in the final systematic Review and Meta-analysis
