# Supplementary material for: Prevalence of depression among medical students in Africa: Systematic review and meta-analysis
Source: PLoS One. 2024 Dec 26;19(12):e0312281. doi: 10.1371/journal.pone.0312281 (PMC11670985; doi:10.1371/journal.pone.0312281)
Supplement: S1 Data — (DOCX) [file pone.0312281.s007.docx]

**Prevention of missing data**

As it known that missing data result in decrease precision and possibly biased of the effect estimates of single studies. However, there was no missing among the included primary articles in our systematic Review and Meta-analysis study because we included them all and pooled estimate was generated. This is because we have an extensive searching of articles in order not to miss data by making our searching and pre-define criteria for including and excluding the primary articles clear and precise. We have used a standardized data extraction form for each single study included and also conducted a robust inspection of our data before formal analysis took over. In addition, we have conducted sensitivity and trim and fill analysis to detect and adjust potential publication bias.
